# Dichotomous keys to the species of *Solanum* L. (Solanaceae) in continental Africa, Madagascar (incl. the Indian Ocean islands), Macaronesia and the Cape Verde Islands

**DOI:** 10.3897/phytokeys.127.34326

**Published:** 2019-07-19

**Authors:** Sandra Knapp, Maria S. Vorontsova, Tiina Särkinen

**Affiliations:** 1 Department of Life Sciences, Natural History Museum, Cromwell Road, London SW7 5BD, UK Natural History Museum London United Kingdom; 2 Comparative Plant and Fungal Biology Department, Royal Botanic Gardens, Kew, Richmond, Surrey TW9 3AE, UK Royal Botanic Gardens Kew United Kingdom; 3 Royal Botanic Garden Edinburgh, 20A Inverleith Row, Edinburgh EH3 5LR, UK Royal Botanic Garden Edinburgh United Kingdom

**Keywords:** Africa, Aldabra, Azores, Canary Islands, Cape Verde, Comoros, cultivated plants, identification, keys, Madagascar, Madeira, Mauritius, La Réunion, Seychelles, *
Solanum
*, weeds

## Abstract

*Solanum* L. (Solanaceae) is one of the largest genera of angiosperms and presents difficulties in identification due to lack of regional keys to all groups. Here we provide keys to all 135 species of *Solanum* native and naturalised in Africa (as defined by World Geographical Scheme for Recording Plant Distributions): continental Africa, Madagascar (incl. the Indian Ocean islands of Mauritius, La Réunion, the Comoros and the Seychelles), Macaronesia and the Cape Verde Islands. Some of these have previously been published in the context of monographic works, but here we include all taxa. The paper is designed to be used in conjunction with the web resource Solanaceae Source (www.solanaceaesource.org) and hyperlinks provide access to online descriptions, synonymy and images (where available) of each species. All taxa treated and specimens seen are included in searchable Suppl. material 1, 2.

## Introduction

*Solanum* L. (Solanaceae) is one of the largest of angiosperm genera (Frodin 2004) with ca. 1,200 species distributed worldwide with species on all continents except Antarctica. The greatest species diversity in the genus occurs in the Neotropics (see Bohs 2005), but significant diversity also occurs in the Old World, with Africa and Australia particularly important areas for diversification (see Vorontsova and Knapp 2016; Echeverría-Londoño et al. 2018). Due to its large number of species and the number of introductions and cultivated taxa, *Solanum* is often an identification challenge for non-specialists. Recent completion of several large monographic treatments of the *Solanum* of Africa (e.g. Vorontsova and Knapp 2016; Knapp and Vorontsova 2016; Särkinen et al. 2018) as part of the US National Science Foundation funded Planetary Biodiversity Inventory project “PBI Solanum” means we can now provide keys for the genus across the continent and for adjacent islands such as Madagascar and Macaronesia. Some of these have been published in the Open Access literature (e.g. Knapp 2013; Knapp and Vorontsova 2016; Särkinen et al. 2018), but the largest of these, treating the spiny solanums (Vorontsova and Knapp 2016) is not. Several species that are either naturalised (e.g. members of the Brevantherum Clade) or cultivated (tree tomatoes, pepinos, potatoes and tomatoes) in the region are also not treated as part of these monographic treatments, although complete species descriptions and photographs are provided on the web resource Solanaceae Source (www.solanaceaesource.org).

**Figure 1.**
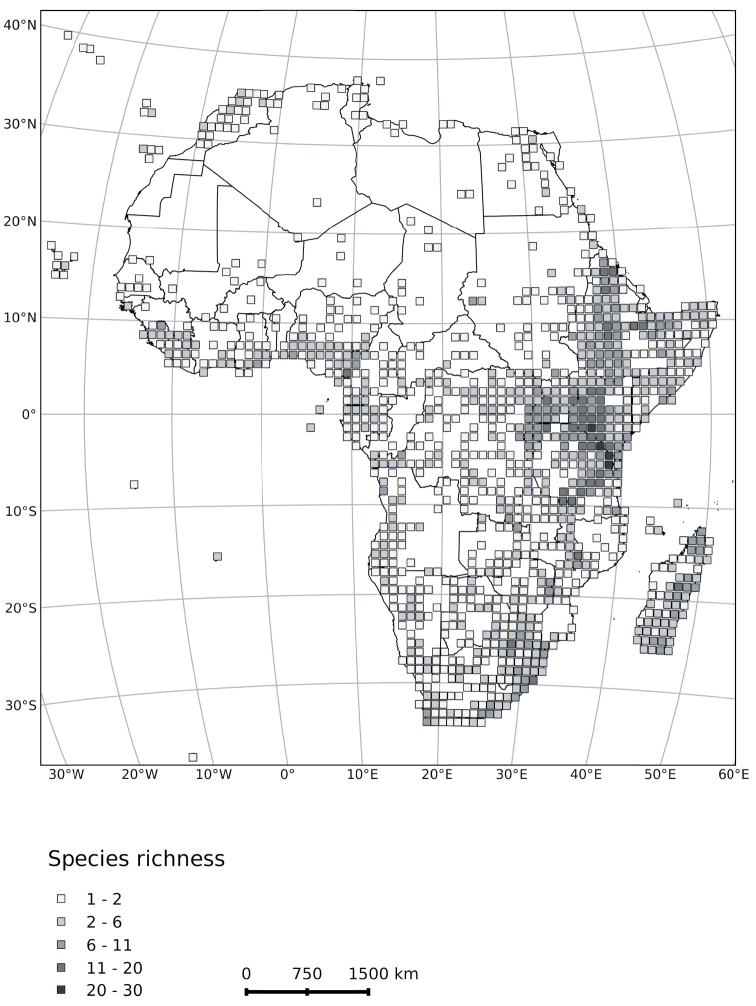
Heat map of *Solanum* diversity in Africa. Darker degree squares indicate greater species richness. The middle to high elevation regions of eastern Africa (Kenya/Tanzania) have the highest high species diversity, followed by secondary areas of species richness in the Ethiopian plateaus, dry areas of central Madagascar, South Africa and the area around Mount Cameroon. We have not analysed how collecting effort has influenced these patterns, but it is likely to be important. As the Leptostemonum Clade has the largest number of species in Africa, diversity in that clade drives species richness overall (see Vorontsova and Knapp 2016, figure 2). Map prepared by Sarah Ficinski.

Here we provide dichotomous keys that include all groups and species (native, naturalised and widely cultivated; see Table 1 for species list) of *Solanum* occurring in continental Africa, Madagascar (incl. the Indian Ocean islands of Mauritius, La Réunion, the Comoros, and the Seychelles), Macaronesia and the Cape Verde Islands to facilitate identification across the region. Taxa occurring in each country in the region are shown in Table 2 and a map of *Solanum* diversity (all taxa) is presented in Figure 1. Keys to individual groups are also provided for the 135 *Solanum* species occurring in the region (see Table 1 and Appendix 1 for a species list). We hope that these keys will encourage collection and documentation of *Solanum* across Africa and uncover new distributions and perhaps new species for the region.

**Table 1.** The 135 species of *Solanum* (native, naturalised and widely cultivated) occurring in Africa as defined by Brummitt (2001), with their places of original publication and clade membership as currently understood (Major Clades sensu Bohs 2005; minor clades are divisions within these sensu Bohs 2007; Stern et al. 2011; Vorontsova et al. 2103; Särkinen et al. 2015, 2018; Aubriot et al. 2016; Tepe et al. 2016).

Species	Place of original publication	Major Clade	Minor clade
*Solanumaculeastrum* Dunal	Prodr. [A. P. de Candolle] 13(1): 366. 1852.	Leptostemonum	Old World-Africa
*Solanumaculeatissimum* Jacq.	Collectanea [Jacquin] 1: 100. 1787 [‘1786’].	Leptostemonum	Acanthophora
*Solanumadoense* Hochst. ex A.Rich.	Tent. Fl. Abyss. 2: 105. 1850 [1851].	Leptostemonum	Old World-Africa
*Solanumaethiopicum* L.	Cent. Pl. 2: 10. 1756.	Leptostemonum	Old World-Africa
*Solanumafricanum* Mill.	Gard. Dict. ed. 8, no. 26. 1768.	African non-spiny (ANS)	
*Solanumagnewiorum* Voronts.	Phytotaxa 10: 32. 2010.	Leptostemonum	Old World-Africa
*Solanumagrarium* Sendtn.	Fl. Bras. (Martius) 10: 68, fig. 5, 32–33. 1846.	Leptostemonum	Gardneri
*Solanumaldabrense* C.H.Wright	Kew Bull. 1894: 149. 1894.	Leptostemonum	Old World
*Solanumamericanum* Mill.	Gard. Dict. ed. 8, no. 5. 1768.	Morelloid	Black nightshade
*Solanumanguivi* Lam.	Tabl. Encycl. 2: 23. 1794.	Leptostemonum	Old World-Africa
*Solanumanomalum* Thonn.	Beskr. Guin. Pl. 126 1827.	Leptostemonum	Old World-Africa
*Solanumarundo* Mattei	Boll. Reale Orto Bot. Giardino Colon. Palermo 7: 188. 1908.	Leptostemonum	Old World-Africa
*Solanumatropurpureum* Schrank	Syll. Ratisb. 1: 200. 1824.	Leptostemonum	Acanthophora
*Solanumaureitomentosum* Bitter	Repert. Spec. Nov. Regni Veg. 11: 18. 1912.	Leptostemonum	Old World-Africa
*Solanumbatoides* D’Arcy & Rakot.	Fl. Madag., Fam. 176: 75. 1994.	Leptostemonum	Old World-Madagascar
*Solanumbetaceum* Cav.	Anales Hist. Nat. 1: 44. 1799.	Cyphomandra	Pachyphylla
*Solanumbetroka* D’Arcy & Rakot.	Fl. Madag., Fam. 176: 77. 1994.	African non-spiny (ANS)	
*Solanumbumeliifolium* Dunal	Prodr. [A. P. de Candolle] 13(1): 292. 1852.	Leptostemonum	Old World-Madagascar
*Solanumburchellii* Dunal	Prodr. [A. P. de Candolle] 13(1): 291. 1852.	Leptostemonum	Old World-Africa
*Solanumcampylacanthum* Hochst. ex A.Rich.	Tent. Fl. Abyss. 2: 102. 1850.	Leptostemonum	Old World-Africa
*Solanumcapense* L.	Syst. ed. 10: 935. 1759.	Leptostemonum	Old World-Africa
*Solanumcapsicoides* All.	Auct. Syn. Meth. Stirp. Hort. Regii Taur. 64. 1773.	Leptostemonum	Acanthophora
*Solanumcatombelense* Peyr.	Sitzungsber. Kaiserl. Akad. Wiss., Math.-Naturwiss. Cl. 38: 576. 1860.	Leptostemonum	Old World-Africa
*Solanumcerasiferum* Dunal	Prodr. [A. P. de Candolle] 13(1): 365. 1852.	Leptostemonum	Old World-Africa
*Solanumchenopodioides* Lam.	Tabl. Encycl. 2: 18. 1794.	Morelloid	Black nightshade
*Solanumchrysotrichum* Schltdl.	Linnaea 19: 304. 1847.	Leptostemonum	Torva
*Solanumcoagulans* Forssk.	Fl. Aegypt.-Arab. 47. 1775.	Leptostemonum	Old World-Africa
*Solanumcordatum* Forssk.	Fl. Aegypt.-Arab. 47. 1775.	Leptostemonum	[not assigned]
*Solanumcroatii* D’Arcy & R.C.Keating	Phytologia 34: 282. 1976.	Leptostemonum	Old World-Madagascar
*Solanumcyaneopurpureum* De Wild.	Pl. Bequaert. 1: 425. 1922.	Leptostemonum	Old World-Africa
*Solanumcymbalariifolium* Chiov.	Boll. Soc. Bot. Ital. 1925: 107. 1925.	Leptostemonum	Old World-Africa
*Solanumdasyphyllum* Schumach. & Thonn.	Beskr. Guin. Pl. 126 [146]. 1827.	Leptostemonum	Old World-Africa
*Solanumdennekense* Dammer	Bot. Jahrb. Syst. 38: 57. 1905.	Leptostemonum	Old World-Africa
*Solanumdiphyllum* L.	Sp. Pl. 184. 1753.	Geminata	
*Solanumelaeagnifolium* Cav.	Icon. 3: 22, tab. 243. 1795.	Leptostemonum	Elaeagnifolium
*Solanumerianthum* D.Don	Prodr. Fl. Nep. 96. 1825.	Brevantherum	
*Solanumerythracanthum* Dunal	Prodr. [A. P. de Candolle] 13(1): 201. 1852.	Leptostemonum	Old World-Madagascar
*Solanumforskalii* Dunal	Hist. Nat. Solanum 237. 1813.	Leptostemonum	Old World-Africa
*Solanumgiganteum* Jacq.	Collectanea [Jacquin] 4: 125. 1791.	Leptostemonum	Old World-Africa
*Solanumglabratum* Dunal	Hist. Nat. Solanum 240. 1813.	Leptostemonum	Old World-Africa
*Solanumgoetzei* Dammer	Bot. Jahrb. Syst. 28: 473. 1900.	Leptostemonum	Old World-Africa
*Solanumguineense* L.	Sp. Pl. 184. 1753.	African non-spiny (ANS)	
*Solanumhastifolium* Hochst. ex Dunal	Prodr. [A. P. de Candolle] 13(1): 284. 1852.	Leptostemonum	Old World-Africa
*Solanumheinianum* D’Arcy & R.C.Keating	Phytologia 34: 282. 1976.	Leptostemonum	Old World-Madagascar
*Solanumherculeum* Bohs	Plant Syst. Evol. 228: 44. 2001.	Normania	
*Solanumhumblotii* Dammer	Bot. Jahrb. Syst. 38: 184. 1906.	African non-spiny (ANS)	
*Solanumhumile* Lam.	Tabl. Encycl. 2: 23. 1794.	Leptostemonum	Old World-Africa
*Solanumimamense* Dunal	Prodr. [A. P. de Candolle] 13(1): 85. 1852.	African non-spiny (ANS)	
*Solanuminaequiradians* Werderm.	Notizbl. Bot. Gart. Berlin-Dahlem 12: 90. 1934.	Leptostemonum	Old World-Africa
*Solanumincanum* L.	Sp. Pl. 188. 1753.	Leptostemonum	Old World-Africa
*Solanuminsanum* L.	Mant. 1: 46. 1767.	Leptostemonum	Old World-Tropical Asia
*Solanumivohibe* D’Arcy & Rakot.	Fl. Madag., Fam. 176: 97. 1994.	African non-spiny (ANS)	
*Solanumjubae* Bitter	Bot. Jahrb. Syst. 54: 501. 1917.	Leptostemonum	Old World-Africa
*Solanumlaciniatum* Aiton	Hort. Kew. ed. 1, 1: 247. 1789.	Archaesolanum	
*Solanumlamprocarpum* Bitter	Repert. Spec. Nov. Regni Veg. Beih. 16: 107. 1923.	Leptostemonum	Old World-Africa
*Solanumlanzae* J.-P.Lebrun & Stork	Candollea 50: 217. 1995.	Leptostemonum	Old World-Africa
*Solanumlichtensteinii* Willd.	Enum. Pl. (Willdenow) 1: 238. 1809.	Leptostemonum	Old World-Africa
*Solanumlidii* Sunding	Blyttia 24: 368. 1966.	Leptostemonum	Old World
*Solanumlinnaeanum* Hepper & P.-M.L. Jaeger	Kew Bull. 41: 435. 1986.	Leptostemonum	Old World-Africa
*Solanumlitoraneum* A.E.Gonç.	Kew Bull. 52(3): 703. 1997.	Leptostemonum	Old World-Africa
*Solanumlycopersicum* L.	Sp. Pl. 185. 1753.	Potato	Tomato
*Solanummacracanthum* A.Rich.	Tent. Fl. Abyss. 2: 106. 1850.	Leptostemonum	Old World-Africa
*Solanummacrocarpon* L.	Mant. Pl. Altera: 205. 1771.	Leptostemonum	Old World-Africa
*Solanummacrothyrsum* Dammer	Bot. Jahrb. Syst. 38: 185. 1906.	African non-spiny (ANS)	
*Solanummadagascariense* Dunal	Prodr. [A. P. de Candolle] 13(1): 99. 1852.	African non-spiny (ANS)	
*Solanummahoriense* D’Arcy & Rakot.	Ann. Missouri Bot. Gard. 73: 498. 1986.	Leptostemonum	Old World-Madagascar
*Solanummalindiense* Voronts.	Syst. Bot. 35: 904. 2010.	Leptostemonum	Old World-Africa
*Solanummammosum* L.	Sp. Pl. 187. 1753.	Leptostemonum	Acanthophora
*Solanummarginatum* L.f.	Suppl. 147. 1781.	Leptostemonum	Old World-Africa
*Solanummauense* Bitter	Repert. Spec. Nov. Regni Veg. Beih. 16: 42. 1923.	Leptostemonum	Old World-Africa
*Solanummauritianum* Scop.	Delic. Fl. Faun. Insubr. 3: 16. 1788.	Brevantherum	
*Solanummelastomoides* C.H.Wright	Bull. Misc. Inform. Kew 1894: 128. 1894.	Leptostemonum	Old World-Africa
*Solanummelongena* L.	Sp. Pl. 186. 1753.	Leptostemonum	Old World-Tropical Asia
*Solanummemphiticum* J.F.Gmel.	Syst. Nat., ed. 13[bis] 2(1): 385. 1791	Morelloid	Black nightshade
*Solanummuricatum* Aiton	Hort. Kew, ed. 1, 1: 250. 1789.	Potato	Basarthrum
*Solanummyoxotrichum* Baker	J. Linn. Soc., Bot. 21: 426. 1885.	Leptostemonum	Old World-Madagascar
*Solanummyrsinoides* D’Arcy & Rakot.	Fl. Madag., Fam. 176: 115. 1994.	African non-spiny (ANS)	
*Solanumnava* Webb & Berthel.	Phyt. Canar. 2. 3(3): 123. 1845.	Normania	
*Solanumnigriviolaceum* Bitter	Repert. Spec. Nov. Regni Veg. Beih. 16: 163. 1923.	Leptostemonum	Old World-Africa
*Solanumnigrum* L.	Sp. Pl. 186. 1753.	Morelloid	Black nightshade
*Solanumnitidibaccatum* Bitter	Repert. Spec. Nov. Regni Veg. 11: 208. 1912.	Morelloid	Black nightshade
*Solanumpampaninii* Chiov.	Res. Sci. Somalia Ital. 1: 128. 1916.	Leptostemonum	Old World-Africa
*Solanumpauperum* C.H.Wright	Bull. Misc. Inform. Kew 1894: 127. 1894.	Leptostemonum	Old World-Africa
*Solanumpectinatum* Dunal	Prodr. [A. P. de Candolle] 13(1): 250. 1852.	Leptostemonum	Lasiocarpa
*Solanumphoxocarpum* Voronts.	Syst. Bot. 35: 903. 2010.	Leptostemonum	Old World-Africa
*Solanumpimpinellifolium* L.	Cent. Pl. 1: 8. 1755.	Potato	Tomato
*Solanumpolhillii* Voronts.	Syst. Bot. 35: 902. 2010.	Leptostemonum	Old World-Africa
*Solanumpseudospinosum* C.H.Wright	Fl. Trop. Afr. [Oliver et al.] 4, 2: 220. 1906.	Morelloid	Black nightshade
*Solanumpyracanthos* Lam.	Tabl. Encycl. 2: 21. 1794.	Leptostemonum	Old World-Madagascar
*Solanumretroflexum* Dunal	Prodr. [A. P. de Candolle] 13(1): 50. 1852.	Morelloid	Black nightshade
*Solanumrichardii* Dunal	Encycl. [J. Lamarck & al.] Suppl. 3: 775. 1814.	Leptostemonum	Old World-Africa
*Solanumrigidum* Lam.	Tabl. Encycl. 2: 23. 1794.	Leptostemonum	Old World-Africa
*Solanumrobustum* H.L.Wendl.	Flora 27: 784. 1844.	Leptostemonum	Erythrotrichum
*Solanumrubetorum* Dunal	Prodr. [A. P. de Candolle] 13(1): 304. 1852.	Leptostemonum	Old World-Africa
*Solanumrunsoriense* C.H.Wright	Uganda Prot. (H.H.Johnston) 1: 326. 1902.	African non-spiny (ANS)	
*Solanumruvu* Voronts.	J. E. Afr. Nat. Hist. 99: 230. (2010) 2011.	Leptostemonum	Old World-Africa
*Solanumsambiranense* D’Arcy & Rakot.	Fl. Madag., Fam. 176: 123. 1994.	African non-spiny (ANS)	
*Solanumsarrachoides* Sendtn.	Fl. Bras. (Martius) 10: 18, tab. 1, fig. 1-8. 1846.	Morelloid	Black nightshade
*Solanumscabrum* Mill.	Gard. Dict. ed. 8, no. 6. 1768.	Morelloid	Black nightshade
*Solanumschimperianum* Hochst. ex A.Rich.	Tent. Fl. Abyss. 2: 98. 1850.	Leptostemonum	Old World-Africa
*Solanumschliebenii* Werderm.	Notizbl. Bot. Gart. Berlin-Dahlem 12: 92. 1934.	Leptostemonum	Old World-Africa
*Solanumschumannianum* Dammer	Pflanzenw. Ost-Afrikas C (Engler): 352. 1895.	Leptostemonum	Old World-Africa
*Solanumsetaceum* Dammer	Pflanzenw. Ost-Afrikas C (Engler): 33. 1895.	Leptostemonum	Old World-Africa
*Solanumsisymbriifolium* Lam.	Tabl. Encycl. 2: 25. 1794.	Leptostemonum	Sisymbriifolium
*Solanumsodomeodes* Kuntze	Revis. Gen. Pl. 3(3): 227. 1898.	Leptostemonum	Old World-Africa
*Solanumsomalense* Franch.	Sert. Somal. 47. 1882.	Leptostemonum	Old World-Africa
*Solanumstipitatostellatum* Dammer	Abh. Königl. Akad. Wiss. Berlin 1894: 63. 1894.	Leptostemonum	Old World-Africa
*Solanumsupinum* Dunal	Prodr. [A. P. de Candolle] 13(1): 289. 1852.	Leptostemonum	Old World-Africa
*Solanumtaitense* Vatke	Linnaea 43: 327. 1882.	Leptostemonum	Old World-Africa
*Solanumtarderemotum* Bitter	Repert. Spec. Nov. Regni Veg. 10: 547. 1912.	Morelloid	Black nightshade
*Solanumterminale* Forssk.	Fl. Aegypt.-Arab. 45. 1775.	African non-spiny (ANS)	
*Solanumtettense* Klotzsch	Naturw. Reise Mossambique (Peters) 1: 237. 1861.	Leptostemonum	Old World-Africa
*Solanumthomsonii* C.H.Wright	Fl. Trop. Afr. [Oliver et al.] 4, 2: 217. 1906.	Leptostemonum	Old World-Africa
*Solanumtoliaraea* D’Arcy & Rakot.	Ann. Missouri Bot. Gard. 76: 351. 1989.	Leptostemonum	Old World-Madagascar
*Solanumtomentosum* L.	Sp. Pl. 188. 1753.	Leptostemonum	Old World-Africa
*Solanumtorreanum* A.E.Gonç.	Kew Bull., 52(3): 706. 1997.	Leptostemonum	Old World-Africa
*Solanumtorvum* Sw.	Prodr. [O. P. Swartz] 47. 1788.	Leptostemonum	Torva
*Solanumtrichopetiolatum* D’Arcy & Rakot.	Fl. Madag., Fam. 176: 130. 1994.	African non-spiny (ANS)	
*Solanumtriflorum* Nutt.	Gen. N. Amer. Pl. 1: 128. 1818.	Morelloid	
*Solanumtrisectum* Dunal	Prodr. [A. P. de Candolle] 13(1): 36. 1852.	Normania	
*Solanumtruncicola* Bitter	Bot. Jahrb. Syst. 54: 435. 1917.	African non-spiny (ANS)	
*Solanumtuberosum* L.	Sp. Pl. 185. 1753.	Potato	Petota
*Solanumumalilaense* Manoko	PhytoKeys 16: 67. 2012.	Morelloid	Black nightshade
*Solanumumtuma* Voronts. & S.Knapp	PhytoKeys 8: 4. 2012.	Leptostemonum	Old World-Africa
*Solanumusambarense* Bitter & Dammer	Repert. Spec. Nov. Regni Veg. Beih. 16: 40. 1923.	Leptostemonum	Old World-Africa
*Solanumusaramense* Dammer	Pflanzenw. Ost-Afrikas C (Engler): 353. 1895.	Leptostemonum	Old World-Africa
*Solanumvespertilio* Aiton	Hort. Kew. ed. 1, 1: 252. 1789.	Leptostemonum	Old World
*Solanumviarum* Dunal	Prodr. [A. P. de Candolle] 13(1): 240. 1852.	Leptostemonum	Acanthophora
*Solanumvillosum* Mill.	Gard. Dict. ed. 8, no. 2. 1768.	Morelloid	Black nightshade
*Solanumviolaceum* Ortega	Nov. Pl. Descr. Dec. 56. 1798.	Leptostemonum	Old World-Tropical Asia
*Solanumvirginianum* L.	Sp. Pl. 187. 1753.	Leptostemonum	Old World-Tropical Asia
*Solanumwendlandii* Hook.f.	Bot. Mag. 113: tab. 6914. 1887.	Wendlandii-Allophyllum	
*Solanumwittei* Robyns	Bull. Jard. Bot. État Bruxelles 17: 82. 1943.	Leptostemonum	Old World-Africa
*Solanumwrightii* Benth.	Fl. Hongk. 243. 1861.	Leptostemonum	Androceras-Crinitum
*Solanumzanzibarense* Vatke	Linnaea 43: 326. 1882.	Leptostemonum	Old World-Africa

**Table 2.** Country distribution of *Solanum* species in Africa (as defined here); introduced (incl. cultivated) species in brackets (epithet); taxa not included in the keys because they are known from a singleton cultivated specimen, are in *italic* type. All records based on specimens examined by the authors with verified identities. The status of *S.torvum* is not completely clear, but it is most likely to be introduced from the New World, so is treated as that here; *S.americanum*, on the other hand, appears to have a worldwide distribution, so is treated as native. The occurrence of *S.rigidum* in Senegal is doubtful, the specimen is very old and the label may be in error. Cultivated plants are often not collected, so the absence of records of commonly cultivated crops (e.g. *S.lycopersicum*, *S.macrocarpon*, *S.tuberosum*) should not be interpreted as lack of occurrence, merely as lack of collections. *Solanumdiphyllum* was recorded from Eygpt by Fawzi and Habeeb (2016) with a verifiable photograph; this Mexican species is widely cultivated and easily naturalised and is likely to be spreading around the Mediterranean.

Country	Species
Algeria	*herculeum*, *linnaeanum*, *nigrum*, *villosum*
Angola	*aculeastrum.* (*aculeatissimum*), *aethiopicum*, *americanum*, *anguivi*, *anomalum*, *aureitomentosum*, (*betaceum*), *campylacanthum*, *capsicoides*, *catombelense*, *dasyphyllum*, *humile*, *lichtensteinii*, (*lycopersicum*), *macrocarpon*, *mammosum*, (*mauritianum*), *pauperum*, *scabrum*, *tarderemotum*, *terminale*, *tettense*, *villosum*
Azores	(*linnaeanum*), (*chenopodioides*), (*chrysotrichum*), (*nava*?), *nigrum*, (*maritianum*), (*pseudocapsicum*), *villosum*
Benin	*anguivi*, *anomalum*, *incanum*, *scabrum*, (*torvum*)
Botswana	*campylacanthum*, *catombelense*, *lichtensteinii*, *retroflexum*, *scabrum*, *supinum*, *tarderemotum*, *tettense*, *villosum*
Burkina Faso	*cerasiferum*, *dasyphyllum*, *incanum*, *scabrum*
Burundi	*aculeastrum*, *anguivi*, *campylacanthum*, *cyaneopurpureum*, *dasyphyllum*, *mammosum*, *memphiticum*, *tarderemotum*, *terminale*, *villosum*
Cabo Verde	(*agrarium*), *americanum*, (*lycopersicum*), *nigrum*, *rigidum*, *scabrum*, *tarderemotum*, (*torvum*)
Cameroon	*aculeastrum*, (*aculeatissimum*), *aethiopicum*, *americanum*, *anguivi*, *anomalum*, *cerasiferum*, *dasyphyllum*, (*erianthum*), *giganteum*, (*lycopersicum*), *macrocarpon*, (*mauritianum*), (*melongena*), *pseudospinosum*, *scabrum*, *tarderemotum*, *terminale*, (*torvum*), (*wendlandii*), (*wrightii*)
Canary Islands (Spain)	*americanum*, (*laxum*), (*lycopersicum*), (*mauritianum*), *nava*, *nigrum*, (*pseudocapsicum*), (*robustum*), *vespertilio*, (*wendlandii*)
Central African Republic (CAR)	*aculeastrum*, (*aculeatissimum*), *anguivi*, *cerasiferum*, *dasyphyllum*, *giganteum*, (*lycopersicum*), *macrocarpon*, *scabrum*, (*seaforthianum*), *terminale*, (*torvum*), (*wrightii*)
Chad	*cerasiferum*, *forskalii*, *incanum*, *tarderemotum*, *villosum*
Comoros (incl. Mayotte)	*americanum*, *macrothyrsum*, *richardii*, *scabrum*, *tarderemotum*, *terminale*, (*torvum*)
Democratic Republic of the Congo	*aculeastrum*, (*aculeatissimum*), *aethiopicum*, *anomalum*, *aureitomentosum*, *campylacanthum*, *cerasiferum*, (*chrysotrichum*), *cyaneopurpureum*, *dasyphyllum*, *giganteum*, *lichtensteinii*, (*lycopersicum*), *macrocarpon*, (*mammosum*), (*mauritianum*), (*melongena*), *memphiticum*, *richardii*, *runsoriense*, *scabrum*, (*seaforthianum*), *tarderemotum*, *terminale*, *tettense*, (*torvum*), (*viarum*), *wittei*, (*wrightii*)
Republic of the Congo	*aculeastrum*, *anomalum*, *dasyphyllum*, (*lycopersicum*), *terminale*, (*torvum*)
Cote d’Ivoire	(*aculeatissimum*), *americanum*, *anguivi*, *anomalum*, *cerasiferum*, *dasyphyllum*, (*lycopersicum*), *scabrum*, *terminale*, (*torvum*)
Djibouti	* somalense *
Egypt (incl. Hala’ib triangle)*	*coagulans*, (*diphyllum*), *dulcamara*, *elaeagnifolium*, *forskalii*, *incanum*, (*lycopersicum*), *macrocarpon*, (*melongena*), *memphiticum*, *nigrum*, *scabrum*, (*torvum*), *villosum*, *virginianum*, (*wendlandii*), (*wrightii*)
Equatorial Guinea	(*aculeatissimum*), *aethiopicum*, *americanum*, *anguivi*, *dasyphyllum*, *giganteum*, (*lycopersicum*), *pseudospinosum*, *scabrum*, *terminale*, (*torvum*)
Eritrea	*adoense*, *americanum*, *anguivi*, *campylacanthum*, *cerasiferum*, *coagulans*, *dasyphyllum*, *forskalii*, *glabratum*, *incanum*, (*lycopersicum*), *macracanthum*, *marginatum*, *melastomoides*, (*melongena*), *memphiticum*, *muricatum*, *scabrum*, *schimperianum*, *somalense*, *tarderemotum*, *terminale*, *villosum*
Ethiopia	(*aculeatissimum*), *adoense*, *americanum*, *anguivi*, *arundo*, *campylacanthum*, *capsicoides*, *cerasiferum*, *coagulans*, *cordatum*, *dennekense*, *forskalii*, *giganteum*, *glabratum*, *hastifolium*, *hirtulum*, *incanum*, *jubae*, *lanzae*, (*lycopersicum*), *macracanthum*, *macrocarpon*, *marginatum*, *melastomoides*, *memphiticum*, *muricatum*, *pampaninii*, *runsoriense*, *schimperianum*, *somalense*, *tarderemotum*, *terminale*, *tettense*, *villosum*, (*wrightii*)
Gabon	*aethiopicum*, *americanum*, *anguivi*, *anomalum*, *dasyphyllum*, *giganteum*, *macrocarpon*, *scabrum*, *terminale*, (*torvum*), (*wrightii*)
Gambia	*americanum*, *anguivi*, *cerasiferum*, *dasyphyllum*
Ghana	(*aculeatissimum*), *americanum*, *anguivi*, *anomalum*, *capsicoides*, *dasyphyllum*, (*erianthum*), *incanum*, *macrocarpon*, (*melongena*), *scabrum*, *tarderemotum*, *terminale*, (*torvum*), (*wrightii*)
Guinea	(*aculeatissimum*), *anguivi*, (*erianthum*), *scabrum*, *tarderemotum*, *terminale*, (*torvum*), (*wrightii*)
Guinea-Bissau	*americanum*, *anguivi*, *cerasiferum*, *dasyphyllum*, *terminale*
Kenya	*aculeastrum*, (*aculeatissimum*), *aethiopicum*, *agnewiorum*, *americanum*, *anguivi*, *arundo*, (*betaceum*), *campylacanthum*, *coagulans*, *cordatum*, *dasyphyllum*, *dennekense*, *forskalii*, *giganteum*, *goetzei*, *hastifolium*, *incanum*, *jubae*, *lanzae*, (*laxum*), (*lycopersicum*), *macrocarpon*, *malindiense*, *mammosum*, *mauense*, (*mauritianum*), *melastomoides*, (*melongena*), *nigriviolaceum*, *pampaninii*, *phoxocarpum*, *polhillii*, (*pseudocapsicum*), *richardii*, *runsoriense*, *schumannianum*, (*seaforthianum*), *setaceum*, *sisymbrifolium*, *somalense*, *stipitatostellatum*, *taitense*, *tarderemotum*, *terminale*, *tettense*, (*tuberosum*), *usambarense*, *usaramense*, *villosum*, (*wendlandii*), (*wrightii*), *zanzibarense*
Lesotho	(*aculeatissimum*), (*chenopodioides*), *lichtensteinii*, *retroflexum*, *scabrum*, *sodomeodes*, *tarderemotum*
Liberia	(*aculeatissimum*), *americanum*, *anguivi*, *anomalum*, *dasyphyllum*, (*lycopersicum*), (*mauritianum*), *scabrum*, *terminale*, (*torvum*)
Libya	*linnaeanum*, *nigrum*, *villosum*, *virginianum*
Madagascar	*aethiopicum*, *americanum*, *anguivi*, *batoides*, (*betaceum*), *betroka*, *bumeliifolium*, *croatii*, *erythracanthum*, *heinianum*, *humblotii*, *imamense*, *insanum*, *ivohibe*, (*lycopersicum*), *macrocarpon*, *madagascariense*, *mahoriense*, (*mauritianum*), (*melongena*), *myoxotrichum*, *myrsinoides*, (*pseudocapsicum*), *pyracanthos*, *richardii*, *sambiranense*, *scabrum*, (*seaforthianum*), *tarderemotum*, *toliaraea*, (*torvum*), *trichopetiolatum*, *truncicola*, (*tuberosum*), *violaceum*
Madeira (Portugal)	(*chenopodioides*), *dulcamara*, (*laxum*), *linnaeanum*, (*lycopersicum*), *marginatum*, *nigrum*, (*pseudocapsicum*), *trisectum*, *villosum*
Malawi	*aculeastrum*, (*aculeatissimum*), *aethiopicum*, *americanum*, *anguivi*, *aureitomentosum*, *campylacanthum*, (*chrysotrichum*), *dasyphyllum*, *giganteum*, *goetzei*, *lichtensteinii*, *macrocarpon*, *retroflexum*, *richardii*, *scabrum*, *schumannianum*, (*seaforthianum*), *tarderemotum*, *terminale*, *tettense*, (*torvum*), *villosum*, (*wendlandii*), (*wrightii*)
Mali	*cerasiferum*, *dasyphyllum*, *forskalii*, *incanum*, (*lycopersicum*), *tarderemotum*
Mauritania	*dasyphyllum*, *scabrum*, *villosum*
Mauritius (incl. La Réunion)	*americanum*, (*anguivi*), (*chenopodioides*), *erythracanthum*, *insanum*, (*lycopersicum*), (*mauritianum*), (*melongena*), *richardii*, *tarderemotum*, (*torvum*), *violaceum*
Morocco	*dulcamara*, *elaeagnifolium*, *forskalii*, *herculeum*, (*laciniatum*), *linnaeanum*, *nigrum*, *triflorum*, *villosum*
Mozambique	*aculeastrum*, (*aculeatissimum*), *aethiopicum*, *americanum*, *anguivi*, *aureitomentosum*, *campylacanthum*, *catombelense*, *dasyphyllum*, *giganteum*, *goetzei*, *lamprocarpum*, *lichtensteinii*, *linnaeanum*, *litoraneum*, *retroflexum*, *richardii*, *scabrum*, *stipitatostellatum*, *tarderemotum*, *tettense*, *torreanum*, (*torvum*), *usaramense*, (*viarum*), *villosum*, *zanzibarense*
Namibia	*burchellii*, *campylacanthum*, *capense*, *catombelense*, *elaeagnifolium*, *numile*, *lichtensteinii*, (*lycopersicum*), *pimpinellifolium*, *retroflexum*, *scabrum*, (*seaforthianum*), *supinum*, *tarderemotum*, *tettense*
Niger	*anguivi*, *forskalii*, *incanum*, (*lycopersicum*), *villosum*
Nigeria	*aculeastrum*, (*aculeatissimum*), *aethiopicum*, *americanum*, *anguivi*, *anomalum*, *cerasiferum*, *dasyphyllum*, (*erianthum*), *giganteum*, *incanum*, (*lycopersicum*), *macrocarpon*, *melongena*, *scabrum*, *terminale*, (*torvum*), *villosum*, (*wrightii*)
Rwanda	*aculeastrum*, (*aculeatissimum*), *anguivi*, *campylacanthum*, *cyaneopurpureum*, *dasyphyllum*, *giganteum*, *tarderemotum*, *terminale*, *wittei*
São Tome e Principe	*americanum*, *capsicoides*, (*melongena*), *scabrum*, *terminale*
Senegal	*anguivi*, *cerasiferum*, *forskalii*, *incanum*, (*lycopersicum*), *rigidum* ?, *scabrum*, *tarderemotum*
Seychelles	*aldabrense*, *americanum*, *scabrum*
Sierra Leone	*aculeatissimum*, *americanum*, *anguivi*, *capsicoides*, *dasyphyllum*, (*erianthum*), (*lycopersicum*), *macrocarpon*, (*melongena*), *scabrum*, *tarderemotum*, *terminale*, (*torvum*), (*wrightii*)
Somalia	*arundo*, *campylacanthum*, *coagulans*, *cordatum*, *cymbalariifolium*, *dasyphyllum*, *dennekense*, *forskalii*, *glabratum*, *hastifolium*, *incanum*, *jubae*, *melastomoides*, (*melongena*), *memphiticum*, *pampaninii*, *schimperianum*, *somalense*, *tarderemotum*, *tettense*, *villosum*
South Africa	*aculeastrum*, (*aculeatissimum*), *africanum*, *americanum*, *anguivi*, *burchellii*, *campylacanthum*, *capense*, *catombelense*, (*chenopodioides*), (*chrysotrichum*), *dasyphyllum*, *elaeagnifolium*, *giganteum*, *guineense*, *humile*, (*laxum*), *lichtensteinii*, *linnaeanum*, (*mauritianum*), (*pseudocapsicum*), *retroflexum*, *rubetorum*, (*sarrachoides*), (*seaforthianum*), *sisymbriifolium*, *sodomeodes*, *supinum*, *tarderemotum*, *terminale*, *tettense*, *tomentosum*, *torreanum*, (*torvum*), *triflorum*, (*viarum*), (*wrightii*)
South Sudan	*aculeastrum*, (*aculeatissimum*), *aethiopicum*, *anguivi*, *campylacanthum*, *cerasiferum*, *coagulans*, *dasyphyllum*, *giganteum*, *hastifolium*, *scabrum*, *tarderemotum*, *terminale*
Sudan (incl. Hala’ib triangle)*	*aculeastrum*, *adoense*, *aethiopicum*, *campylacanthum*, *cerasiferum*, *coagulans*, *forskalii*, *hastifolium*, *incanum*, *macrocarpon*, *memphiticum*, *nigrum*, *scabrum*, *schimperianum*, *somalense*, *tarderemotum*, *villosum*
Swaziland	*aculeastrum*, *campylacanthum*, *catombelense*, *retroflexum*, (*robustum*), (*seaforthianum*), *sisymbriifolium*, *torreanum*
Tanzania	*aculeastrum*, (*aculeatissimum*), *americanum*, *anguivi*, *arundo*, (*atropurpureum*), *aureitomentosum*, (*betaceum*), *campylacanthum*, *coagulans*, *cyaneopurpureum*, *dasyphyllum*, *dennekense*, *giganteum*, *goetzei*, *hastifolium*, *inaequiradians*, *lamprocarpum*, *lanzae*, *lichtensteinii*, (*lycopersicum*), *macrocarpon*, *mauense*, (*melongena*), *memphiticum*, (*pectinatum*), *phoxocarpum*, *polhillii*, *richardii*, (*robustum*), *scabrum*, *schliebenii*, *schumannianum*, (*seaforthianum*), *setaceum*, *stipitatostellatum*, *taitense*, *tarderemotum*, *terminale*, *tettense*, *thomsonii*, (*tuberosum*), *umalilaense*, *usambarense*, *usaramense*, *villosum*, (*wendlandii*), *wittei*, (*wrightii*), *zanzibarense*
Togo	(*aculeatissimum*), *aethiopicum*, *americanum*, *anguivi*, *anomalum*, (*melongena*), *scabrum*, *terminale*, (*torvum*)
Tunisia	*linnaeanum*, (*lycopersicum*), *nigrum*, *triflorum*, *villosum*
Uganda	*aculeastrum*, (*aculeatissimum*), *aethiopicum*, *americanum*, *anguivi*, (*betaceum*), *campylacanthum*, *cerasiferum*, *coagulans*, *cyaneopurpureum*, *dasyphyllum*, *giganteum*, *hastifolium*, *lanzae*, *macrocarpon*, *mammosum*, *mauense*, *mauritianum*, *memphiticum*, *runsoriense*, *scabrum*, *schumannianum*, (*seaforthianum*), *tarderemotum*, *terminale*, *tettense*, *villosum*, *wittei*, (*wrightii*)
Western Sahara	* villosum *
Zambia	(*aculeatissimum*), *americanum*, *anguivi*, *aureitomentosum*, *campylacanthum*, (*chrysotrichum*), *goetzei*, *lichtensteinii*, (*lycopersicum*), *retroflexum*, *richardii*, *scabrum*, (*seaforthianum*), *tarderemotum*, *terminale*, *tettense*, (*torvum*), (*tuberosum*), *villosum*, (*wendlandii*), (*wrightii*)
Zimbabwe	*aculeastrum.* (*aculeatissimum*), *anguivi*, *aureitomentosum*, (*betaceum*), *campylacanthum*, *catombelense*, *giganteum*, *lichtensteinii*, *linnaeanum*, (*mauritianum*), *retroflexum*, *richardii*, *scabrum*, (*seaforthianum*), *tarderemotum*, *terminale*, *villosum*, (*wendlandii*)

*Possession of the area known as the Hala’ib triangle is disputed between Egypt and Sudan, species occurring there are listed under both coutries.

## Materials and methods

We modified keys from published monographs for groups of *Solanum* from the botanical continent “Africa” as defined in the World Geographical Scheme for Recording Plant Distributions (WGSRPD; Brummitt 2001). This corresponds basically to the countries of the continent of Africa, but excludes the Sinai Peninsula (politically part of Egypt and in WGSRPD part of Western Asia). It also includes islands grouped as Macaronesia (Azores, Canary Islands, Madeira and the Cape Verde Islands ) and Madagascar and other Indian Ocean islands east to Rodrigues.

We assessed distribution using the published monographs, with additional data points added from subsequent herbarium visits. All specimens seen for these keys can be seen in the Supplemental File and in the dataset published on the NHM Data Portal (https://doi.org/10.5519/0042549). For descriptions of the taxa, users are referred to the original publications or the Solanaceae Source website (www.solanaceaesource.org), where all species treated here are described and synonymy listed.

To access descriptions on the Solanaceae Source website, begin by typing the species name in the search box in the upper right-hand part of the screen banner (tick the option “Taxonomy” below the box); when the correct name you are searching for appears, select it, then push the “Search” button to the right of the search box (if you do not push the “Search” button, nothing will happen). You will be taken to the species page, where images and synonyms appear on the opening page; to access descriptions, click on the “Description” tab where information can be obtained. Up-to-date specimen details are not currently available on the website but can be found as described above.

## Keys

**Figure 2.**
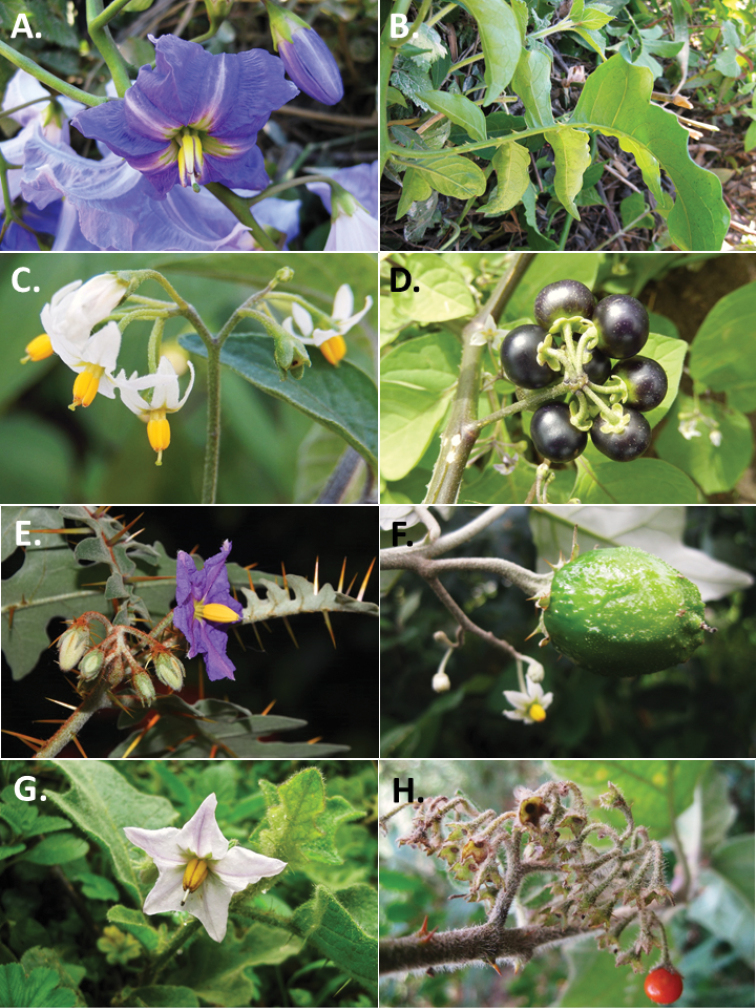
**A, B***Solanumwendlandii* Hook.f. (Allophyllum-Wendlandii Clade) **C***Solanumtarderemotum* Bitter (Morelloid Clade) **D***Solanumscabrum* Mill. (Morelloid Clade) **E***Solanumpyracanthos* Lam. (Leptostemonum Clade) **F***Solanumaculeastrum* Dunal (Leptostemonum Clade) **G***Solanumnigriviolaceum* Bitter (Leptostemonum Clade) **H***Solanumusambarense* Bitter & Dammer (Leptostemonum Clade). Photos **A, B, F, G H** by M.S. Vorontosova **C, D, E** by S. Knapp.

**Figure 3.**
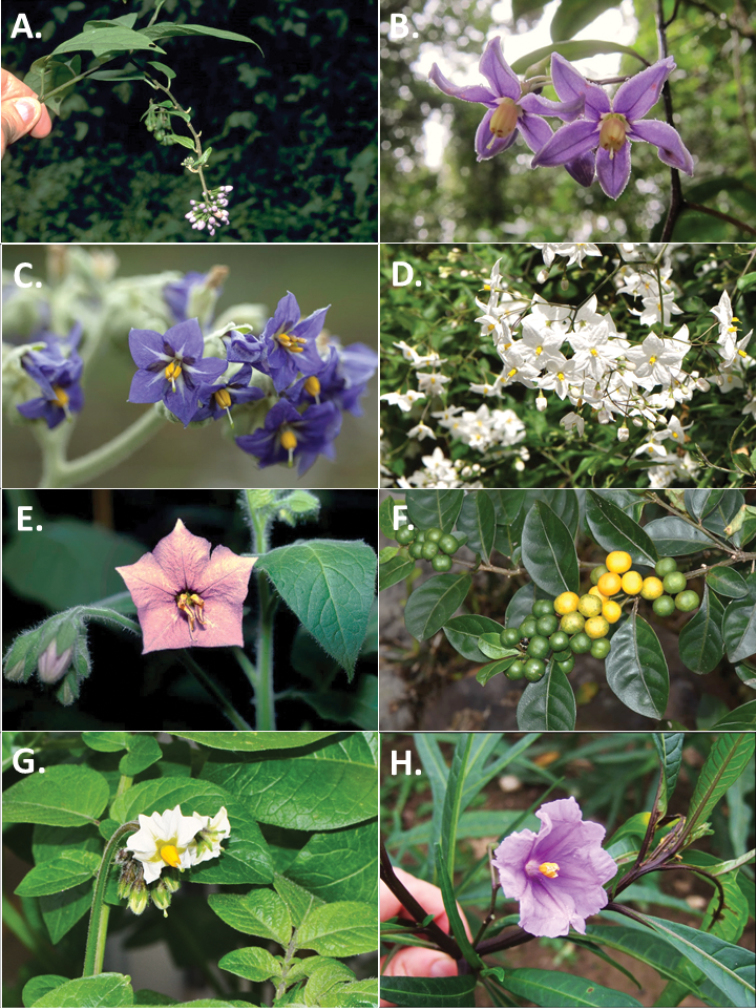
**A***Solanumterminale* Forssk. (African non-spiny Clade) **B***Solanummadagascariense* Dunal (African non-spiny Clade) **C***Solanummauritianum* Scop. (Brevantherum Clade) **D***Solanumlaxum* Spreng. (Dulcamaroid Clade) **E***Solanumtrisectum* Dunal (Normania Clade) **F***Solanumdiphyllum* L. (Geminata Clade) **G***Solanumtuberosum* L. (Potato Clade) **H***Solanumlaciniatum* Aiton (Archaeosolanum Clade). Photos **A, C, D, E, F, G, H** by S. Knapp **B** by M.S. Vorontsova.

*Solanum* can be divided into 13 major clades or monophyletic groups (Bohs 2005; Weese and Bohs 2007; Särkinen et al. 2013; see Figures 2 and 3 for photographs illustrating representative morphology of these groups in Africa). The largest monophyletic clade is the Leptostemonum clade, or the “spiny solanums”, which comprises approximately half of the species diversity of the genus; divisions within that clade have been defined by Stern et al. (2011), Vorontsova et al. (2013) and Aubriot et al. (2016). This group is rapidly diversifying in the Old World (Echeverría-Londoño et al. 2018), with most taxa occurring in the Old World belonging to a single monophyletic group. Previous treatments (e.g. Whalen 1984; Jaeger 1985; Jaeger and Hepper 1986) had suggested the African taxa were members of, or closely related to New World groups. More information on the phylogenetic relationships of African and Asian members of the Leptostemonum Clade can be found in Vorontsova et al. (2013) and Aubriot et al. (2016). Other clades with significant species diversity in Africa (as defined here) are the African non-spiny (ANS) and Normania Clades (both endemic to the region; see Bohs and Olmstead 2001) and the Morelloid Clade (with a number of widespread weedy taxa, see Särkinen et al. 2018). Other clades such as the Geminata, Brevantherum and Potato Clades are represented only by introduced or cultivated species. The Dulcamaroid Clade has a single species native to Mediterranean northern Africa and Macaronesia and two cultivated taxa that can become naturalised (Knapp 2013). In order to facilitate identification and to assist with the discovery of novelties from the region, we provide a key to the major groups (clades) of *Solanum* following the most recent phylogeny of the genus (Särkinen et al. 2013) and additional dichotomous keys to the species within each group. Groups are ordered as they occur as branches in the phylogeny of Särkinen et al. (2013).

Four species have been recorded from this area, for which we have only seen single specimens, all of which are cultivated and not naturalised. *Solanumlaciniatum* Aiton (Archaesolanum Clade), the kangaroo apple from Australia and New Zealand, has been recorded from Morocco, *S.agrarium* Sendtn. (Leptostemonum Clade, sectionAcanthophora Dunal sensu Nee 1979) from Brazil has only recently been collected in the Cape Verde Islands and *S.atropurpureum* Schrank (Leptostemonum Clade, sectionAcanthophora Dunal sensu Nee 1979) from Brazil and *S.pectinatum* Dunal (Leptostemonum Clade, sectionLasiocarpa Dunal sensu Whalen et al. 1981) from Mexico have been recorded from Tanzania in a botanical garden. These singletons have been included in Table 1, but not in the keys below; descriptions should be checked if identification is ambiguous.

Each species name is hyperlinked to its page on Solanaceae Source (www.solanaceaesource.org) where photographs (if available), descriptions and other information can be found. An expanded key to all of the thirteen major clades of *Solanum* worldwide is in preparation (R. Hilgenhof and T. Särkinen, pers. comm.). Instructions on how to use Solanaceae source are included in the Materials and Methods.

### Key to the groups of *Solanum* in continental Africa, Madagascar (incl. the islands of Réunion, the Comoros and the Seychelles), Macaronesia and the Cape Verde Islands

**Table:** 

1a	Trichomes of stems and leaves stellate or echinoid	**2**
1b	Trichomes of stems and leaves simple (unbranched) or dendritically branched, never stellate or echinoid	**3**
2a	Anthers ellipsoid in outline; inflorescences many times branched; branching dichasial; stems without prickles	**Key 6. Brevantherum Clade**
2b	Anthers tapering in outline; inflorescences branched or unbranched; branching monochasial; stems with or without prickles	**Key 7. Leptostemonum Clade**
3a	Shrubs, small trees or woody vines	**4**
3b	Herbs or if plants woody, only at the base; never true vines, occasionally scandent	**9**
4a	Stems with hooked prickles; anthers tapering	***Solanumwendlandii* Hook.f. (Wendlandii-Allophyllum Clade)**
4b	Stems without prickles; anthers ellipsoid	**5**
5a	Small trees with foetid cordate leaves; flowers waxy pink or greenish white; anther connectives enlarged; fruit a large turbinate berry with fleshy pulp; cultivated tree tomato	***Solanumbetaceum* Cav. (Pachyphylla [Cyphomandra] Clade)**
5b	Shrubs or woody vines; leaves, flowers and anther connectives not as above	**6**
6a	Small shrubs with paired (geminate) leaves; flowers nodding, white; fruit held on erect pedicels, orange; cultivated or occasionally naturalised in northern Africa	**7**
6b	Woody vines or scandent shrubs; leaves, flowers and fruit not as above; continental Africa, Madagascar; or cultivated	**8**
7a	Leaves lanceolate, with at least some branched trichomes on new growth; leaves of a pair more or less the same shape; inflorescence usually with 2 flowers; berry dark orange; cultivated “Jerusalem cherry”	***Solanumpseudocapsicum* L. (Geminata Clade)**
7b	Leaves ovate or elliptic, completely glabrous; leaves of a pair markedly different in shape; inflorescence with more than 2 flowers; berry pale orange; only recorded from Egypt	***Solanumdiphyllum* L. (Geminata Clade)**
8a	Base of pedicel enclosed in a small sleeve of rhachis tissue usually more than 0.5 mm long; Mediterranean northern Africa; Macaronesia; if in other parts of Africa, cultivated	**Key 3. Dulcamaroid Clade**
8b	Base of pedicel peg-like, sometimes enclosed in a small sleeve of rhachis tissue, if so the sleeve less than 0.5 mm long; Continental tropical Africa; Madagascar; native plants	**Key 1. African non-spiny (ANS) Clade**
9a	Leaves pinnate or deeply pinnatifid	**11**
9b	Leaves simple (at most the margins toothed) or at most ternate	**12**
10a	Fleshy prostrate herbs; leaves pinnatifid, the leaflets not distinct; inflorescences unbranched	***Solanumtriflorum* Nutt. (Morelloid Clade)**
10b	Spreading herbs or herbaceous scramblers, not fleshy; leaves pinnate with distinct leaflets; inflorescences branched or less often unbranched	**Key 5. Potato Clade**
11a	Anthers dimorphic, of different sizes and two of the five with horn-like projections; Macaronesia and northern Africa	**Key 2. Normania Clade**
11b	Anthers equal in size and shape, if unequal only slightly so; widespread or cultivated	**12**
12a	Trichomes simple with a single long terminal cell (bayonet hairs); fruit a large greenish berry with purple stripes (more than 3 cm diameter), with abundant solid mesocarp; herbaceous vine	***Solanummuricatum* Aiton (Potato Clade)**
12b	Trichomes simple or branched; fruit variously coloured (usually less than 1 cm in diameter), with juicy mesocarp; annual or short-lived perennial herbs	**Key 4. Morelloid Clade**

### Key 1. African non-spiny (ANS) Clade (descriptions Knapp and Vorontsova 2016)

**Table:** 

1a	Leaves glabrous on both surfaces	**2**
1b	Leaves with at least some pubescence on either surface (this sometimes sparse along veins and midrib)	**10**
2a	Inflorescence few-flowered, unbranched (at most furcate in *Solanumbetroka*)	**3**
2b	Inflorescence many flowered, usually many times branched	**5**
3a	Flowers appearing fasciculate and axillary; corolla usually somewhat campanulate; fruit orange; South Africa	***Solanumguineense* L.**
3b	Flowers not appearing fasciculate; corolla stellate, the petals spreading or reflexed; fruit colour green, black or not known, never orange; Madagascar	**4**
4a	Leaves clustered on short shoots; calyx lobes deltate, not divided to base; dry forests	***Solanumbetroka* D’Arcy & Rakot.**
4b	Leaves not clustered on short shoots; calyx lobes long triangular, divided to the base; wet forests	***Solanumtruncicola* D’Arcy & Rakot.**
5a	Flowers or fruits (or pedicel scars) in tightly packed groups on individual branches (these sometimes very short and the inflorescence appearing spicate)	***Solanumterminale* Forssk.**
5b	Flowers spaced on the open inflorescence, often unevenly so	**6**
6a	Leaves clustered on short shoots	***Solanumbetroka* D’Arcy & Rakot.**
6b	Leaves spaced along the stem	**7**
7a	Anthers opening by pores that elongate with age; mountains of continental Africa	***Solanumrunsoriense* C.H.Wright**
7b	Anthers opening by delineated pores that do not elongate with age; Madagascar	**8**
8a	Leaves fleshy, thick and coriaceous, the venation not visible in dry specimens; fruit with thick pericarp (woody?)	***Solanummyrsinoides* D’Arcy & Rakot.**
8b	Leaves membranous to coriaceous, not markedly thick and fleshy, the venation visible in dry specimens; fruit with thin pericarp, the seeds visible through the berry wall	**9**
9a	Petioles with long, simple trichomes (these not extending to the lamina); seeds 4–8 per berry; inflorescence axis thin and delicate	***Solanumtrichopetiolatum* D’Arcy & Rakot.**
9b	Petioles glabrous or with minute dendritic trichomes; seeds 20–40 per berry; inflorescence axis robust	***Solanummadagascariense*** Dunal
10a	Leaf trichomes simple (unbranched)	**11**
10b	Leaf trichomes branched (dendritic to echinoid)	**16**
11a	Inflorescence axis unbranched, the flowers closely spaced	**12**
11b	Inflorescence axis branched, often many times so	**13**
12a	Leaves clustered along stem; fruit orange; South Africa	***Solanumguineense* L.**
12b	Leaves spaced along shoots; fruit purple or black; Madagascar	***Solanumtruncicola* D’Arcy & Rakot.**
13a	Flowers or fruits (or pedicel scars) in tightly packed groups on individual branches (these sometimes very short and the inflorescence appearing spicate)	***Solanumterminale* Forssk.**
13b	Flowers spaced on the open inflorescence, often unevenly so	**14**
14a	Stems strongly quadrangular; at least some leaves with shallow lobes; plants of seashore and dune habitats	***Solanumafricanum* Mill.**
14b	Stems terete; leaves not lobed; plants of forests and forest edges	**15**
15a	Leaf pubescence very sparse, confined to the midrib or near the petiole; flowers not heterostylous; Madagascar	***Solanumtrichopetiolatum* D’Arcy & Rakot.**
15a	Leaf pubescence variable, not very sparse, along veins and lamina; flowers heterostylous; mountains of continental Africa	***Solanumrunsoriense* C.H.Wright**
16a	Abaxial leaf surfaces with tufts of trichomes in the vein axils (domatia)	**17**
16b	Abaxial leaf surfaces with trichomes on lamina and/or along veins, not with prominent tufts in the vein axils (domatia)	**19**
17a	Inflorescence many times branched, open and with many flowers (more than 20); calyx lobes broadly deltate; petioles to 4 cm long, thin and flexuous; Mayotte (Comoros)	***Solanummacrothyrsum* Dammer**
17b	Inflorescence furcate, more congested and with fewer flowers (fewer than 20); calyx lobes deltate; petioles to 2.5 cm long, thicker; Madagascar	**18**
18a	Calyx lobes 0.8–2 mm long; inflorescences with 10–16 flowers	***Solanumivohibe* D’Arcy & Rakot.**
18b	Calyx lobes 4–6 mm long; inflorescences with 3–10 flowers	***Solanumsambiranense* D’Arcy & Rakot.**
19a	Abaxial leaf surfaces evenly pubescent on veins and lamina	**20**
19b	Abaxial leaf surfaces pubescent only along the veins and midrib, the trichomes not extending to the lamina	**22**
20a	Anther pores lengthening to slits with age; flowers heterostylous; leaves evenly distributed along branches; mountains of continental Africa	***Solanumrunsoriense* C.H.Wright**
20b	Anther pores not lengthening to slit with age; flowers not heterostylous; leaves usually at least somewhat clustered on short shoots; Madagascar	**21**
21a	Leaves densely pubescent with golden (when dry) loosely dendritic trichomes; flowers more than 2 cm in diameter; anthers 4–6 mm long; widespread in Madagascar	***Solanumimamense* Dunal**
21b	Leaves sparsely pubescent with white (when dry) congested dendritic trichomes; flowers 2 cm in diameter or less; anthers 3.5–4 mm long; dry forests of southern Madagascar	***Solanumbetroka* D’Arcy & Rakot.**
22a	Inflorescence unbranched, with few flowers; pedicels 1.8–4.5 cm long	***Solanumhumblotii* Bitter**
22b	Inflorescence many times branched, with many flowers; pedicels 0.8–1.2 cm long	**23**
23a	Anther pores lengthening to slits with age; flowers heterostylous; pedicels with pubescence like the inflorescence rhachis; mountains of continental Africa	***Solanumrunsoriense* C.H.Wright**
23b	Anther pores not lengthening to slit with age; flowers not heterostylous; pedicels always glabrous; Madagascar	***Solanummadagascariense* Dunal**

### Key 2. Normania clade (descriptions on Solanaceae Source)

**Table:** 

1a	Leaves shallowly lobed, pubescent with long, tangled eglandular trichomes; anthers tapering, horned near the base, tightly connivent; seeds more than 5 mm long; fruit a dry berry; Mediterranean	***Solanumherculeum* Bohs**
1b	Leaves simple or ternate, glabrous or pubescent, but the trichomes not long and tangled, glandular; anthers markedly horned, spreading; seeds less than or equal to 5 mm long; fruit a brightly coloured, juicy berry; laurisylva forest in Macaronesia	**2**
2a	Leaves simple or ternate, the base truncate or cordate if leaves unlobed; anthers yellow, horned in lower third; berry bright red; Madeira	***Solanumtrisectum* Dunal**
2b	Leaves simple, the base cordate; anthers black, horned about halfway up from the base; berry orange or red; Tenerife, Canary Islands	***Solanumnava* Webb & Berthel.**

### Key 3. Dulcamaroid clade (descriptions in Knapp 2013 and on Solanaceae Source)

**Table:** 

1a	Buds turbinate and strongly pointed; petals strongly reflexed, with shiny green dots at the base of each; anthers tightly connivent with “glue”; fruit a shiny red berry, often ellipsoid; native plants in Mediterranean northern Africa	***Solanumdulcamara* L.**
1b	Buds rounded, often somewhat inflated; petals spreading, without shiny green dots; anthers not tightly connivent with “glue”; fruit red or black, globose; cultivated plants, occasionally naturalised throughout the region	**2**
2a	Flowers white; anthers on equal filaments; leaves with axillary tufts of trichomes on the lower surfaces (domatia), usually simple, occasionally pinnatifid; berry (very rarely) black	***Solanumlaxum* Spreng.**
2b	Flowers purple; one filament slightly longer than the other 4; leaves completely glabrous, pinnatifid, rarely simple; berry bright shiny red	***Solanumseaforthianum* Andrews**

### Key 4. Morelloid clade (descriptions in Särkinen et al. 2018 and on Solanaceae Source)

**Table:** 

1a	Leaves shallowly to deeply pinnatifid	***Solanumtriflorum* Nutt.**
1b	Leaves entire to sinuate-dentate	**2**
2a	Glandular trichomes present (e.g. along stems, petioles and leaves), plants usually sticky to touch when fresh	**3**
2b	Glandular trichomes absent (e.g. along stems, petioles and leaves), plants not sticky to touch when fresh	**14**
3a	Anthers less than 1.8 mm long	**4**
3b	Anthers more than or equal to 1.8 mm long	**7**
4a	Inflorescences with 10–40 flowers; pedicels spaced 1–2 mm apart, sharply bent at the base (near articulation point) in flower and fruit	***Solanumtarderemotum* Bitter**
4b	Inflorescences with 2–5(-10) flowers; pedicels spaced 0–1 mm apart, nodding, erect or spreading in flower and fruit, reflexed and slightly curved in some species in fruit but never in flower	**5**
5a	Calyx lobes 1–1.5 mm long in flower; fruiting calyces not accrescent, the tube remaining 1–1.7 mm long and lobes 1–1.5 mm long; fruit black when ripe, not markedly shiny, with a glaucous cast	***Solanumretroflexum* Dunal**
5b	Calyx lobes 1.5–2.5 mm long in flower; fruiting calyces accrescent, the tube 3–4 mm long and lobes 2.5–8.0 mm long; fruit green when ripe, shiny	**6**
6a	Leaf bases attenuate to cuneate; inflorescences mostly intermodal, with 4–8(-10) flowers; pedicels spaced 0.3–1 mm apart; calyx lobes 1.7–2.5 mm long; corollas with yellow-green central eye with black-purple V-shaped margins; anthers 1.0–1.4 mm long; berries dark green to green-brown marbled with white lines, becoming usually translucent and glossy, lower half of berries covered with enlarged calyces but berry mostly visible; seeds brown; stone cells (1-)2–3, these 0.5 mm in diameter; northern Africa	***Solanumnitidibaccatum* Bitter**
6b	Leaf bases truncate; inflorescences mostly leaf-opposed, with 2–5(-7) flowers; pedicels spaced 0(-1) mm apart; calyx lobes 1.5–2.0 mm long; corolla with yellow-green or translucent basal star without black-purple colouration; anthers 1.2–2.0 mm long; berries pale green, shiny becoming dull, opaque, usually completely enveloped by enlarged calyces; seeds pale yellow; stone cells 4–6, these (0.5-)0.8–1 mm in diameter; only known from South Africa	***Solanumsarrachoides* Sendtn.**
7a	Anthers more than or equal to 2.8 mm long	**8**
7b	Anthers less than 2.8 mm long	**9**
8a	Inflorescences with bracteoles present in most individuals; buds narrowly ellipsoid; corolla deeply stellate, the lobes narrowly lanceolate; berries with more than 30 stone cells	***Solanumtriflorum* Nutt.**
8b	Inflorescences never with bracteoles; buds globose, ovoid or narrowly ellipsoid; corolla rotate-stellate, the lobes long- triangular with rounded tips; berries with (0-)2–4 stone cells	***Solanummemphiticum* Forssk.**
9a	Calyx lobes appressed to spreading in fruit, never strongly reflexed	**10**
9b	Calyx lobes strongly reflexed in fruit	**12**
10a	Calyx accrescent in fruit, calyx tube 3–4 mm long and lobes 2.5–8 mm long	***Solanumsarrachoides* Sendtn.**
10b	Calyx not accrescent in fruit, calyx tube 1–2 mm long and lobes 1–1.5 mm long	**11**
11a	Buds ellipsoid; calyx tube 1.5–2.0 mm long, lobes 1–1.5 mm long, elongate-deltate with rounded tips; fruiting pedicels persist when fruits mature and fall off; Cameroon line (Cameroon and Equatorial Guinea), above 2,000 m elevation	***Solanumpseudospinosum* C.H.Wright**
11b	Buds subglobose; calyx tube 0.8–1.0 mm long, lobes 0.5–0.8 mm long, triangular with rounded to acute tips; fruiting pedicels generally do not persist and fall off with maturing fruits; in continental Africa only in South Africa and around the Mediterranean	***Solanumnigrum* L.**
12a	Leaves rhomboidal to lanceolate; filaments 1.2–1.5 mm long, anthers 1.3–1.8(-2) mm long; seeds 1.6–1.8 mm long and 1.3–1.5 mm wide	***Solanumretroflexum* Dunal**
12b	Leaves broadly to narrowly ovate to elliptic; filaments 0.5–1.3 mm long; anthers 1.8–2.5 mm long; seeds 1.8–2.2 mm long and 1.5–1.7 mm wide	**13**
13a	Calyx with broad and relatively transparent sinuses, lobes elliptic to triangular, rounded at tip; free part of the filaments 1.0–1.3 mm long; mature berries slightly ellipsoid, shiny yellow, orange or red; stone cells always absent	***Solanumvillosum* Mill.**
13b	Calyx with narrow, sharp triangular sinuses, lobes deltate with acute or rounded tips; free part of the filaments 0.5–0.7 mm long; mature berries round, dull black or green; stone cells 0–4; in Africa only in South Africa and around the Mediterranean	***Solanumnigrum* L.**
14a	Anthers less than 1.8 mm long	**15**
14b	Anthers more than or equal to 1.8 mm long	**17**
15a	Pedicels spaced 1–2 mm apart, pedicels sharply bent at the base (near the articulation point) in flower and fruit	***Solanumtarderemotum* Bitter**
15b	Pedicels spaced 0–0.5 mm apart, pedicels nodding, erect or spreading in flower and fruit	**16**
16a	Leaves with entire margins, occasionally sinuate-dentate; calyx lobes 0.3–0.5 mm long in flower, 1(-2) mm in fruit; mature fruits black, the surface very shiny	***Solanumamericanum* Mill.**
16b	Leaves shallowly toothed, occasionally entire; calyx lobes 1.0–1.5 mm long in flower, 1.5–2 mm in fruit; mature fruits purple-black or green, the surface dull	***Solanumretroflexum* Dunal**
17a	Anthers less than 2.8 mm long	**18**
17b	Anthers more than or equal to 2.8 mm long	**27**
18a	Berries without stone cells	**19**
18b	Berries with 2–22 stone cells	**24**
19a	Pedicels persisting and not dropping with mature fruits; calyx lobes in fruit mostly strongly reflexed	**20**
19b	Pedicels dropping with mature fruits; calyx lobes in fruit appressed to slightly spreading, rarely strongly reflexed	**22**
20a	Leaves rhomboidal to lanceolate; filaments 1.2–1.5 mm long, anthers 1.3–1.8(-2) mm long; seeds 1.6–1.8 mm long and 1.3–1.5 mm wide	***Solanumretroflexum* Dunal**
20b	Leaves broadly to narrowly ovate to elliptic; filaments 0.5–1.3 mm long; anthers 1.8–2.5 mm long; seeds 1.8–2.2 mm long and 1.5–1.7 mm wide	**21**
21a	Calyx with broad and relatively transparent sinuses, lobes elliptic to triangular, rounded at tip; filaments 1.0–1.3 mm long; mature berries slightly ellipsoid, shiny yellow, orange or red; stone cells always absent	***Solanumvillosum* Mill.**
21b	Calyx with narrow, sharp triangular sinuses, lobes deltate with acute tips; filaments 0.5–0.7 mm long; mature berries round, dull black or green; stone cells generally absent (2–4 stone cells common in Asian material)	***Solanumnigrum* L.**
22a	Buds elongate-oblong; fruiting peduncles strongly deflexed at the base (bent downwards at junction with the stem)	***Solanumchenopodioides* Lam.**
22b	Buds ellipsoid to subglobose; fruiting peduncles straight or ascending	**23**
23a	Pedicels spaced 1–2 mm apart, sharply bent at the base (near the articulation point) in flower and fruit; seeds 1.5–2 mm long and 1–1.5 mm wide	***Solanumtarderemotum* Bitter**
23b	Pedicels spaced 0–0.7 mm apart, straight, spreading or reflexed in flower and fruit; seeds 1.8–2 mm long and 1.5–1.6 mm wide	***Solanumnigrum* L.**
24a	Prostrate herb; leaves narrowly elliptic to lanceolate, base strongly attenuate; inflorescences with 1–5 flowers; pedicels stout and spreading; calyx lobes linear-oblong with rounded apices; mountains of Ethiopia	***Solanumhirtulum* C.H.Wright**
24b	Upright or spreading herb; leaves broadly ovate to elliptic, base acuminate, acute, obtuse, truncate to abruptly attenuate; inflorescences with 2–40 flowers; pedicels thinner, spreading to strongly reflexed; calyx lobes triangular, broadly deltoid or ovate with acute to rounded apices	**25**
25a	Pedicels strongly bent downwards at the base (near articulation point) in flower and fruit	***Solanumtarderemotum* Bitter**
25b	Pedicels spreading, stout or pendent in flower, occasionally recurved in fruit but never strongly bent downwards at the base	**26**
26a	Inflorescences unbranched or more often branched, often with small leaves (bracteoles?); calyx lobes broadly deltate to mere enations of the rim; style exserted 1.0–1.5 mm beyond anther cone; mature berries 3–4(-5) mm in diameter, dull yellowish brown	***Solanumumalilaense* Manoko**
26b	Inflorescences unbranched, never with small leaves; calyx lobes triangular; style exserted 0–1 mm beyond anther cone; mature berries 6–10 mm in diameter, dull black	***Solanumnigrum* L.**
27a	Inflorescences with bracteoles present in most individuals; buds narrowly elliptic; berries with more than 30 stone cells	***Solanumtriflorum* Nutt.**
27b	Inflorescences never with bracteoles; buds globose, ovoid or narrowly ellipsoid; berries with 0–14 stone cells	**28**
28a	Berries with 2–14 stone cells; leaf base strongly attenuate	***Solanumhirtulum* C.H.Wright**
28b	Berries without stone cells; leaf base not strongly attenuate	**29**
29a	Buds elongate-oblong; calyx lobes broadly deltate to triangular with acute tips; fruiting peduncles strongly bent at the base near junction with the stem; fruiting pedicels thin, reflexed and slightly recurved; seeds 1.2–1.4 mm long and 1.0–1.2 mm wide	***Solanumchenopodioides* Lam.**
29b	Buds globose-subglobose; calyx lobes broadly deltate with rounded tips; fruiting peduncles straight; fruiting pedicels stout, erect and spreading; seeds 2–2.8 mm long and 1.5–1.8 mm wide	***Solanumscabrum* Mill.**

### Key 5. Potato clade (descriptions on Solanaceae Source)

**Table:** 

1a	Flowers yellow; anthers tightly connivent and tapering to a beak-like tip; fruit a bright red berry; cultivated tomatoes	**2**
1b	Flowers white or purple; anthers ellipsoid, not tapering to a beak-like tip; fruit green or whitish green (often with purple stripes)	**3**
2a	Corolla lobes deltate to triangular; anther cone stout, the style included; berry usually more than 1 cm in diameter (often much larger), fewer than 20 per infructescence; leaflets with serrate margins; cultivated tomato	***Solanumlycopersicum* L.**
2b	Corolla lobes narrowly triangular; anther cone long and narrow, the style exserted; berry less than 1 cm in diameter, more than 20 per infructescence; leaflets with entire margins; cultivated currant tomato	***Solanumpimpinellifolium* L.**
3a	Leaves at most ternate, usually simple; fruit a berry more than 3 cm in diameter; plant not bearing underground tubers; cultivated pepino	***Solanummuricatum* Aiton**
3b	Leaves pinnate; fruit a berry less than 3 cm in diameter; plant bearing underground tubers; cultivated potato	***Solanumtuberosum* L.**

### Key 6. Brevantherum clade (descriptions on Solanaceae Source)

**Table:** 

1a	Young flower buds turbinate; calyx densely pubescent within over entire surface; young stems sulcate; axillary leaves absent	***Solanumerianthum* D.Don**
1b	Young flower buds oblong to orbicular; calyx lobes pubescent within only in distal quarter; young stems terete; axillary leaves common	***Solanummauritianum* Scop.**

### Key 7. Leptostemonum clade (descriptions in Vorontsova and Knapp 2016 and on Solanaceae Source)

**Table:** 

1a	Young stems and petioles noticeably winged; mature fruit densely pubescent; invasive plant in Tanzanian highlands and South Africa	***Solanumrobustum* H.Wendl.**
1b	Young stems and petioles not markedly winged (terete or slightly ridged); mature fruit glabrous; native or invasive, widespread	**2**
2a	Flowers with stamens of differing lengths (due either to unequal anthers or unequal filaments or both); arid eastern and north-eastern Africa	**3**
2b	Flowers with all stamens equal in length; widespread	**8**
3a	Corolla strongly zygomorphic, with the two lower lobes much larger; flowers often enantiostylous; Canary Islands	**4**
3b	Corolla only weakly zygomorphic, the lower lobes somewhat but not markedly larger; flowers not enantiostylous; continental Africa	**5**
4a	Leaves narrowly elliptic to lanceolate; calyx lobes linear and awn-like; corolla always 5-merous; ripe berry orange; Gran Canaria	***Solanumlidii* Sunding**
4b	Leaves ovate; calyx lobes linear; corolla often 4-merous; ripe berry yellow or yellowish green; Tenerife and Gran Canaria	***Solanumvespertilio* Aiton**
5a	Leaves orbicular to reniform, 1.2–2.5 cm long, wider than long; petioles longer than leaves; rare in north-eastern Somalia	***Solanumcymbalariifolium* Chiov.**
5b	Leaves ovate to elliptic or lanceolate, 2–14 cm long, longer than wide; petioles shorter than leaves; arid eastern and north-eastern Africa	**6**
6a	Stem prickles dense, acicular, less than 0.5 mm wide at base, pale yellow; fruit fully concealed by the accrescent calyx	***Solanumcoagulans* Forssk.**
6b	Stem prickles absent or sparse, if present wider than 1 mm at base, yellow to orange or brown; fruit at least partly exposed	**7**
7a	Leaves subentire to lobed; anthers of equal length; seeds very dark brown to almost black	***Solanummelastomoides* C.H.Wright**
7b	Leaves entire; one anther much longer than the others; seeds dull yellow to orange-brown	***Solanumsomalense* Franch.**
8a	Flower one per inflorescence, peduncle and rhachis absent; corolla pentagonal, lobed for 1/4–1/3 of the way to the base, 0.9–1.3 cm in diameter; southern Africa	***Solanumsupinum* Dunal**
8b	Flower usually more than one per inflorescence, peduncle and/or rhachis present in at least some inflorescences; corolla usually stellate, lobed for more than 1/3 of the way to the base or, if lobed, for 1/4–1/3 of the way to the base then corolla of long-styled flowers broader than 1.3 cm in diameter; widespread	**9**
9a	Trichomes on young stems and adaxial (upper) surfaces of the leaves simple only, never stellate	**10**
9b	Trichomes on young stems and adaxial (upper) surfaces of the leaves stellate	**13**
10a	Flowers pale bluish purple; anthers 8–12.5 mm; fruit globose or extended into a “nipple”	***Solanummammosum* L.**
10b	Flowers white or greenish white; anthers 5–7.5 mm; fruit globose	**11**
11a	No stellate hairs anywhere on the plant; fruit bright orange at maturity; seeds winged, 4–6 mm long	***Solanumcapsicoides* All.**
11b	Stellate hairs almost always present on the abaxial (lower) surface of the leaves; fruit yellow at maturity; seeds not winged, 1.8–2.8 mm long	**12**
12a	Leaf lobes 2–3 pairs, extending 1/3–1/2 distance to the midvein; calyx lobes 5–6.5 mm long, often caudate	***Solanumaculeatissimum* Jacq.**
12b	Leaf lobes 3–5 pairs, extending less than 1/3 of the distance to the midvein; calyx lobes 0.8–2 mm long, acute	***Solanumviarum* Dunal**
13a	Leaves entire, 3–10 times longer than wide; shrubs erect, 1–6 m tall; stem trichomes with partly fused rays; southern Madagascar	**14**
13b	Leaves entire or lobed, 1–3(8) times longer than wide; shrubs erect, scandent or climbing, 0.2–6 m tall; if leaves entire and more than 3 times longer than wide, then shrub less than 1 m tall and not in southern Madagascar; stem trichomes with free rays	**16**
14a	Leaves 9–13(20) cm long; corolla 2–3.1 cm in diameter; juvenile branches with dark red prickles; south-eastern Madagascar	***Solanumcroatii* D’Arcy & Keating**
14b	Leaves 1.5–7 cm long; corolla 1–2 cm in diameter; juvenile branches with grey-brown or red-brown prickles; south-western Madagascar	**15**
15a	Leaf blades 3–7 cm long, concolorous to weakly discolorous, yellow-green	***Solanumbumeliifolium* Dunal**
15b	Leaf blades 1.5–3(4) cm long, strongly discolorous, green-brown adaxially and glaucous abaxially	***Solanumheinianum* D’Arcy & Keating**
16a	Prickles and leaf venation noticeably dark orange to red, contrasting with the yellow-green to red-green leaf surface; southern Madagascar	***Solanumpyracanthos* Lam.**
16b	Prickles and leaf venation not a contrasting colour, yellow to green or red-brown; widespread or naturalised	**17**
17a	Mature fruit green, never developing to bright yellow, orange or red; plants weakly andromonoecious; fruits 1–1.5 cm in diameter	**18**
17b	Mature fruit yellow, orange or red; plants hermaphroditic or andromonoecious, fruits 0.5–6 cm in diameter, if andromonoecious, then fruits more than 1.5 cm in diameter	**19**
18a	Shrub to tree 1.5–9 m tall; young stems and leaves densely ferruginous pubescent; trichomes on the inflorescences and pedicels not glandular; prickles straight to slightly curved	***Solanumchrysotrichum* Schltdl.**
18b	Shrub to 3 m; young stems and leaves pubescent green to brownish; trichomes on the inflorescences and pedicels glandular; prickles curved	***Solanumtorvum* Sw.**
19a	Mature fruit yellow or greenish yellow, 1.5–5(6) cm in diameter; corolla on long-styled flowers (1.3)2–6 cm in diameter; plants mostly andromonoecious	**20**
19b	Mature fruit orange to red, 0.5–1.2(1.7) cm in diameter; if mature fruit orange (1)1.5–2.5(5) cm in diameter, the plant cultivated; corolla on long-styled flowers 0.8–3 cm in diameter; plants mostly hermaphroditic	**45**
20a	Cultivated tree 5–10 m tall with copious flowers; corolla bright purple aging to white with both colours usually present in an inflorescence, 5.5–8 cm in diameter	***Solanumwrightii* Benth.**
20b	Wild plants or cultivated vegetables, less than 6 m tall; corolla white to mauve or purple with the colour constant within each individual, 1.6–6 cm in diameter	**21**
21a	Petiole usually decurrent, leaf bases attenuate (cuneate); trichomes on abaxial leaf surface (if present) stalked with 4(5) rays	**22**
21b	Petiole never decurrent, leaf bases cordate to cuneate; trichomes on abaxial leaf surface (if present) sessile or stalked with 6–16 rays	**23**
22a	Plant clearly stellate-pubescent and armed, drying yellow-green to red-brown; wild plant	***Solanumdasyphyllum* Schumach, & Thonn.**
22b	Plant usually glabrous and unarmed, drying a distinctive red-brown colour; cultivated plant	***Solanummacrocarpon* L.**
23a	Climbers or scandent plants	**24**
23b	Plants erect or rarely semi-scandent	**27**
24a	Prickles on young stems straight	**25**
24b	Prickles on young stems strongly curved	**26**
25a	Prickles pale straw-yellow, to 20 mm long; petiole with sessile stellate trichomes; corolla ca. 2.5 cm in diameter; style strongly curved; Mediterranean northern Africa (adventive from Asia)	***Solanumvirginianum* L.**
25b	Prickles yellow (but not straw-coloured) or brown; petiole trichomes usually stalked; corolla 3.5–5 cm in diameter; style straight; southern Kenya	***Solanumnigriviolaceum* Bitter**
26a	Corolla white, 1.3–1.6 cm in diameter; seeds 5.5–6 mm long; Kenyan mountains	***Solanumagnewiorum* Voronts.**
26b	Corolla mauve to purple, 3.5–6 cm in diameter; seeds 3–4 mm long; eastern and southern Africa, Madagascar	***Solanumrichardii* Dunal**
27a	Calyx inflated, fully covering the berry at maturity; young stems densely covered with straight prickles; northern Madagascar	***Solanummahoriense* D’Arcy & Rakot.**
27b	Calyx not inflated, the berry exposed at maturity; young stems prickly or unarmed; widespread	**28**
28a	Corolla lobed for more than half way to the base; shrubs or trees up to 6 m; variety of habitats	**29**
28b	Corolla lobed for half way to the base or less; small shrubs up to 2 m; weeds of open arid environments or cultivated crops; relatives of the eggplant	**35**
29a	Leaves 8–18 cm long, strongly discolorous; young fruits plain green; seeds 3.5–4.5 mm long; wet upland habitats	**30**
29a	Leaves 2–8 cm long, concolorous or sometimes discolorous; young fruits striped in different shades of green; seeds 2.2–3.5 mm long; arid upland or lowland habitats	**32**
30a	Fruit globose, never apiculate, 1.4–1.7 cm in diameter, 4–10 per infructescence; young stems with yellow (when dry) long-stalked trichomes, the stalks 1–3 mm	***Solanumthomsonii* C.H.Wright**
30b	Fruit turbinate or cone-shaped, sometimes globose, usually somewhat apiculate, 2.8–5 x 1.8–4.5 cm, 1–3(5) per infructescence; young stems usually lacking long-stalked yellow (when dry) trichomes	**31**
31a	Fruit distinctly turbinate or cone-shaped, 2.8–3.7 x 1.8–2.2 cm; leaves on fertile branches elliptic and subentire, 6–8 x 2.5–4 cm, ca. 2.5 times longer than wide; 2100–3000 m elevation	***Solanumphoxocarpum* Voronts.**
31b	Fruit globose, usually somewhat apiculate, 3–5 x 2–4.5 cm; leaves on fertile branches ovate(elliptic) and lobed(subentire), 8–15 x 6–12 cm, 1.5–2 times longer than wide; 1200–2100(3200) m elevation	***Solanumaculeastrum* Dunal**
32a	Prickles straight or occasionally curved; petioles 1/3–2/3 as long as the leaf blades	***Solanumpolhillii* Voronts.**
32b	Prickles on young stems strongly curved; petioles less than 1/3 of the leaf blade length	**33**
33a	Leaves entire, densely stellate-pubescent on both sides; eastern and north-eastern African highlands	***Solanumdennekense* Dammer**
33b	Leaves lobed, adaxially glabrescent; eastern and north-eastern Africa	**34**
34a	Leaves 2–4 cm long; curved prickles on young stems 5–10 mm long; eastern and north-eastern Africa	***Solanumarundo* Matthei**
34b	Leaves 6–8 cm long; curved prickles on young stems 1–3 mm long; coastal Kenya, rare	***Solanummalindiense* Voronts.**
35a	Fruit with soft pericarp, in a variety of shapes and colours, edible; common fasciation in the flowers (e.g. increase in the number of flower parts up to 8), inflated ovaries; cultivated species	***Solanummelongena* L.**
35a	Fruit spherical, yellow, with comparatively hard pericarp, not edible; flowers 5-merous; wild plants	**36**
36a	Leaves lobed with primary and secondary lobes, the primary lobes extending 2/3–3/4 of the distance to the midvein and secondary lobes always present; southern Africa and northern African coasts around the Mediterranean	***Solanumlinnaeanum* Hepper & P.M-L.Jaeger**
36b	Leaves entire or lobed, lobes extending up to 2/3 of the distance to the midvein, secondary lobes usually not present; widespread	**37**
37a	Leaf margins and venation nearly white and contrasting with greenish red-brown adaxial leaf surface; trichomes on the abaxial surface of the leaves with 10–17 rays; Ethiopian highlands	***Solanummarginatum* L.f.**
37b	Leaf margins and venation the same colour as the rest of the leaf blade; trichomes on the abaxial surface of the leaves with 5–12(15) rays; widespread	**38**
38a	Leaf lobes apically obtuse to acute, sometimes rounded, sometimes with secondary lobes; lobes 1/4–2/3 of the distance to the midvein; leaves and young stems glabrescent to moderately pubescent	**39**
38b	Leaf lobes apically rounded, sometimes obtuse, never with secondary lobes; lobes up to 1/3(1/2) of the distance to the midvein; leaves and young stems usually densely pubescent	**41**
39a	Calyx lobes on long-styled flowers 7–10 mm long, ovate and foliaceous, apically obtuse; South Africa	***Solanumumtuma* Voronts. & S.Knapp**
39b	Calyx lobes on long-styled flowers 4–7 mm long, deltate or long-triangular apically acuminate; northern Africa and Cape Verde Islands	**40**
40a	Calyx lobes on long-styled flowers 4–7 mm long, deltate, ca. 1/6 as long as the fruit at maturity; continental Africa north of the equator	***Solanumcerasiferum* Dunal**
40b	Calyx lobes on long-styled flowers 6–7 mm long, long triangular, 1/2 to 1/3 as long as the fruit at maturity; Cape Verde Islands (Senegal?)	***Solanumrigidum* Lam.**
41a	Prickles straight; corolla on long-styled flowers 1.8–2.5 cm in diameter; anthers ca. 4.5 mm long; Madagascar; Mauritius, Réunion	***Solanuminsanum* L.**
41b	Prickles curved or straight; corolla on long-styled flowers 2.5–4.5 cm in diameter; anthers 5–9 mm long; widespread	**42**
42a	Leaves usually entire, sometimes lobed; trichomes on the abaxial leaf surface sessile or with stalks up to 0.1 mm long; fruits 1.5–3 cm diameter	***Solanumcampylacanthum* Hochst. ex A.Rich.**
42b	Leaves lobed; trichomes on the abaxial leaf surface with stalks up to 0.5(1) mm long; fruits 2.5–4.5 cm diameter	**43**
43a	Leaves velvety red-brown adaxially; calyx lobes on long-styled flowers ovate to oblong, foliaceous, 7–10 mm long	***Solanumaureitomentosum* Bitter**
43b	Leaves yellow-green to green-brown adaxially; calyx lobes on long-styled flowers deltate, usually not foliaceous, 2.5–6 mm long	**44**
44a	Leaves concolorous to weakly discolorous, indumentum yellowish; leaves ca. 1.5 times longer than wide; young stems terete to angular; north-eastern Africa	***Solanumincanum* L.**
44b	Leaves strongly discolorous, indumentum dirty greenish brown adaxially and whitish yellow abaxially; leaves 1.5–2.5 times longer than wide; young stems with somewhat raised longitudinal ridges; southern Africa	***Solanumlichtensteinii* Willd.**
45a	Leaves on fertile branches with distinct lobes, at least some of the lobes longer than 1/4 of the distance from the midvein to the leaf edge	**46**
45b	Leaves on fertile branches entire or subentire or with some shallow lobes no longer than 1/4 of the distance from the midvein to the leaf edge	**89**
46a	Leaves more than 3 times longer than wide	**47**
46b	Leaves 1–3 times longer than wide	**49**
47a	Plant unarmed or prickles straight, orange to red; trichomes on the abaxial surfaces of the leaves with (9)12–14 rays	***Solanumelaeagnifolium* Cav.**
47b	Plant densely armed with curved broad-based pale-yellow prickles; trichomes on the abaxial surfaces of the leaves, if present, with 0–8 rays	**48**
48a	Anthers 2.5–3.2 mm long; trichomes anywhere on the plant with 0–4 rays; South Africa	***Solanumsodomaeodes* Kuntze**
48b	Anthers 5–6.5 mm long; trichomes anywhere on the plant with 5–8 rays; north-eastern Africa	***Solanumglabratum* Dunal**
49a	Leaves with at least some secondary lobing present	**50**
49b	Leaves lobed once only	**51**
50a	Flowers 3–7 per inflorescence, mauve to purple; anthers 5.5–7 mm long; South Africa	***Solanumrubetorum* Dunal**
50b	Flowers 6–50 per inflorescence, white; anthers 9–10 mm long; invasive in South Africa and Kenya	***Solanumsisymbriifolium* Lam.**
51a	No prickles visible anywhere on the plant	**52**
51b	At least some prickles visible on the plant	**57**
52a	Plant scandent or scrambling, sometimes erect; anthers (4)5–6.5 mm long; inland eastern Africa	***Solanumcyaneopurpureum* De Wild.**
52b	Plant erect; anthers 2.3–5(8.5) mm long; widespread	**53**
53a	Corolla 1.5–3 cm in diameter; mauve or purple; southern Africa, Indian Ocean islands	**54**
53b	Corolla 0.9–1.5 cm in diameter; white (only occasionally pale violet); widespread on continent or cultivated	**55**
54a	Fruiting pedicels deflexed, curved; anthers 3.5–5.2 mm long; South Africa and Namibia	***Solanumburchellii* Dunal**
54b	Fruiting pedicels straight and spreading; anthers 4.5–8.5 mm long; Mauritius and Réunion	***Solanumviolaceum* Ortega**
55a	Inflorescences 2.5–6 cm long, with 5–22 flowers	***Solanumanguivi* Lam.**
55b	Inflorescences 1–2.5 cm long, with 1–4(10) flowers	**56**
56a	Leaf blades 5–18 cm long, petioles 1–4 cm long; plant cultivated for leaves or fruits, widespread	***Solanumaethiopicum* L.**
56b	Leaf blades 3–8 cm long, petioles 0.5–1.5 cm long; wild plant in southern Africa	***Solanumcatombelense* Peyr.**
57a	Prickles on young stems predominantly curved	**58**
57b	Prickles on young stems predominantly straight, sometimes straight and reflexed	**70**
58a	Young prickles and indumentum red; scandent shrubs; Madagascar	**59**
58b	Young prickles and indumentum yellowish, green or brown; erect or scandent shrubs; widespread (mainland Africa, rarely Madagascar, Indian Ocean Islands)	**60**
59a	Young stems with a dense covering of long-stalked trichomes and prickles of different lengths; 1000–3000 m elevation	***Solanummyoxotrichum* Baker**
59b	Young stems with a sparse or dense covering of sessile or short-stalked trichomes of uniform lengths; 0–1000 m elevation	***Solanumerythracanthum* Dunal**
60a	Stem trichomes with stalks 0.1–0.4 mm long; anthers 6–10 mm long; coastal eastern Africa	***Solanumusaramense* Dammer**
60b	Stem trichomes sessile or the stalks to 0.1 mm long; anthers 3–8.5 mm long; widespread	**61**
61a	Plants of the Indian Ocean islands (Mauritius, Réunion, Aldabra)	**62**
61b	Plants of continental Africa	**63**
62a	Fruiting pedicels straight and spreading; corolla to 3 cm in diameter; petiole not winged from decurrent leaf base; Mauritius and Réunion	***Solanumviolaceum* Ortega**
62b	Fruiting pedicels curved and deflexed; corolla to 2 cm in diameter; petiole slightly winged from decurrent leaf base; Aldabra (Seychelles)	***Solanumaldabrense* C.H.Wright**
63a	Calyx in flower 7–9 mm long, apically caudate; calyx in fruit 10–12 mm long; trichomes with midpoints much longer that the rays; Tanzanian highlands, rare	***Solanuminaequiradians* Bitter**
63b	Calyx in flower 2–6 mm long, apically acute to acuminate or rarely caudate; calyx in fruit less than 10 mm long; trichomes with the midpoints short than or equal to the rays; widespread	**64**
64a	Plant always erect; leaves discolorous; corolla 0.8–1.5 cm in diameter, lobed for 1/2–2/3(3/4) of the way to the base	**65**
64b	Plant often scandent or semi-scandent; leaves usually concolorous; corolla (1)1.5–2.5 cm in diameter, lobed for 2/3 of the way to the base or more	**66**
65b	Inflorescences 2.5–6 cm long, with 5–22 flowers; polymorphic weed across highland areas of Africa and Madagascar	***Solanumanguivi* Lam.**
65a	Inflorescences 1–2.5 cm long, with 1–4 flowers; southern Africa	***Solanumcatombelense* Peyr.**
66a	Prickles 3–5 mm long; leaf blades 1.5–2.5(9) cm long, leaf base narrow-cuneate to attenuate; South Africa and Namibia	***Solanumcapense* L.**
66b	Prickles 1–4(7) mm long. Leaf blades (1)2.5–14 cm long, leaf base cuneate to truncate; widespread	**67**
67a	Calyx 2–4 mm long in flower	**68**
67b	Calyx 4–6 mm long in flower	**69**
68a	Prickles on young stems 1.5–2(3) mm long; leaves 2.5–5.5 cm long; seeds 2.5–3.5 mm long; inland eastern Africa	***Solanumcyaneopurpureum* De Wild.**
68b	Prickles on young stems 2–4 mm long. Leaves 3–14 cm long; seeds 1.8–2.5 mm long; coastal eastern Africa	***Solanumzanzibarense* Vatke**
69a	Leaves drying yellow-green; petiole 1/4–1/3 of the leaf length; eastern and north-eastern Africa	***Solanumhastifolium* Hochst. ex Dunal**
69b	Leaves drying dark red-brown; petiole 1/3–1/2 of the leaf length; southern Africa	***Solanumtorreanum* A.E.Gonç.**
70a	Inflorescence with more than 10 flowers	**71**
70b	Inflorescence with 10 or fewer flowers	**74**
71a	Leaves 1–4(6) cm long; corolla 1.3–2.4 cm in diameter; seeds almost black; north of the equator	***Solanumforskalii* Dunal**
71b	Leaves (5)7–25 cm long; corolla 0.7–1.5 cm in diameter; seeds yellow to orange-brown; widespread	**72**
72a	Inflorescences 1–2.5 cm long, peduncle 0–2 mm long; trichomes on the young stems with (5)8–20 rays; western Africa	***Solanumanomalum* Thonn.**
72b	Inflorescences 2.5–8 cm long, peduncle 2–30 mm long; trichomes on the young stems with 6–8 rays; widespread	**73**
73a	Inflorescences branched many times (more than once); Kenya and Tanzania	***Solanumusambarense* Bitter**
73b	Inflorescences unbranched or forked; widespread	***Solanumanguivi* Lam.**
74a	Young prickles almost filiform and easily bendable like bristles, with no firm prickles anywhere on the plant; eastern African savannah	***Solanumsetaceum* Dammer**
74b	Young prickles sturdy when touched; widespread	**75**
75a	Calyx lobes oblong, foliaceous, apically obtuse; lowland eastern Africa, rare	***Solanumlamprocarpum* Bitter**
75b	Calyx lobes deltate, sometimes long-ovate or narrow-oblong, not clearly foliaceous, apically acute to acuminate; widespread	**76**
76a	Calyx 7–12 mm long in flower	**77**
76b	Calyx 2–7 mm long in flower	**80**
77a	Prickles on young branches 8–16 mm long; leaves elliptic; Ethiopian highlands	***Solanummacracanthum* A.Rich.**
77b	Prickles on young branches 2–7 mm long; leaves ovate; Tanzania, South Africa and Madagascar	**78**
78a	Corolla lobed for 3/4 of the way to the base or more; trichomes on the young stems with midpoints (0.5)1.5–2.5(3) mm long; Tanzanian highlands	***Solanuminaequiradians* Bitter**
78b	Corolla lobed for 1/2–2/3 of the way to the base; trichomes on the young stems with midpoints 0.15–5 mm long; South Africa and Madagascar	**79**
79a	Young stems with a dense covering of long-stalked trichomes and prickles of different lengths; Madagascar	***Solanummyoxotrichum* Baker**
79b	Young stems with no bristles, only prickles and trichomes of uniform length; South Africa	***Solanumtomentosum* L.**
80a	Young prickles and indumentum red; pedicels (1)1.5–4 cm long; Madagascar	**81**
80b	Young prickles and indumentum pale yellow to brown or purple, never red; pedicels 0.2–1(1.4) cm long; mainland Africa, rarely in Madagascar	**82**
81a	Young stems with a dense covering of long-stalked trichomes and prickles of different lengths; 1000–3000 m elevation	***Solanummyoxotrichum* Baker**
81b	Young stems with a sparse or dense covering of sessile or short-stalked trichomes of uniform lengths; 0–1000 m elevation	***Solanumerythracanthum* Dunal**
82a	Corolla lobed for 3/4 of the way to the base or deeper; plants scrambling or climbing, sometimes erect	**83**
82a	Corolla lobed for 1/2–2/3 of the way to the base; plants erect, sometimes scandent	**86**
83a	Prickles on young stems 2–4 mm long; eastern African coastal areas	***Solanumzanzibarense* Vatke**
83b	Prickles on young stems (3)4–13 mm long; arid environments across continental Africa	**84**
84a	Prickles reflexed; seeds nearly black	***Solanumforskalii* Dunal**
84b	Prickles perpendicular to the stem; seeds pale yellow to orange-brown	**85**
85a	Leaves 1–2 times longer than wide; leaf lobes to 2/3 of the distance to the midvein; leaves concolorous to strongly discolorous; north-eastern Africa	***Solanumadoense* Hochst. ex A. Rich.**
85b	Leaves 1.5–3 times longer than wide; leaf lobes to 1/2 of the distance to the midvein; leaves concolorous; South Africa	***Solanumhumile* Dunal**
86a	Petiole 1/3–2/3 as long as the leaf blade; South Africa	***Solanumtomentosum* L.**
86b	Petiole 1/6–1/3 as long as the leaf blade; widespread	**87**
87a	Leaf blades (5)11–25 cm long; inflorescence with 5–22 flowers; common variable highland weed widespread across Africa	***Solanumanguivi* Lam.**
87b	Leaf blades 1.7–9 cm long; inflorescence with 1–6 flowers; southern Africa	**88**
88a	Leaves elliptic; corolla 1.5–2.2 cm in diameter, mauve to purple	***Solanumburchellii* Dunal**
88b	Leaves ovate, sometimes oblong; corolla 0.9–1.3 cm in diameter, usually white	***Solanumcatombelense* Peyr.**
89a	At least some inflorescences branched several to many times; inflorescence with (10)20–150 flowers; erect shrubs to small trees	**90**
89b	Inflorescences unbranched or forked; inflorescence with 1–10(20) flowers; scrambling, scandent or erect shrubs	**98**
90a	Plants with at least some visible prickles or bristles	**91**
90b	Plants with no prickles or bristles visible	**95**
91a	Young stems with soft or thin bristles	**92**
91b	Young stems with firm deltate prickles	**93**
92a	Stem bristles soft and white, 0.4–0.7 mm wide at base; leaves ca. 2 times longer than wide; 1500 -1800 m elevation; Tanzania	***Solanumschliebenii* Werderm.**
92b	Stem bristles erect purple-black or brown, 0.2–0.4 mm wide at base; leaves 2.5–3.5 times longer than wide; 1800–2600 m elevation; eastern Africa	***Solanumschumannianum* Dammer**
93a	Leaf blades 12–40 cm long, whitish grey underneath with trichomes, falling as white powder when touched; inflorescences with 30–150 flowers; anthers 2.5–3 mm long	***Solanumgiganteum* Jacq.**
93b	Leaf blades 4–10(16) cm long, yellow-green, brown or rarely whitish grey underneath, trichomes not falling when touched; inflorescences with 10–30 flowers; anthers 3.5–6 mm long	**94**
94a	Leaf base cuneate to acuminate; anthers 3.5–4 mm long; stem trichomes with midpoints 0.4–1(2) mm long; Democratic Republic of the Congo, Rwanda, Uganda, Tanzania; rare	***Solanumwittei* Robyns**
94b	Leaf base rounded, rarely cuneate; anthers 4–6 mm long; stem trichomes with midpoints 0.05–0.2 mm long; widespread	***Solanumtettense* Klotzsch**
95a	Leaves elliptic, with 8–12 pairs of primary veins, 2.5–3.5 times longer than wide	***Solanumschumannianum* Dammer**
95b	Leaves ovate, sometimes elliptic, with 4–8 pairs of primary veins, 1.5–2.5(3) times longer than wide	**96**
96a	Leaves concolorous; calyx lobes 2–4(5) mm long, long-acuminate; Sudan, Eritrea, Ethiopia, Somalia, 1500–2800 m elevation	***Solanumschimperianum* Hochst. ex A.Rich.**
96b	Leaves discolorous; calyx lobes 1–2 mm long, obtuse to acute, sometimes acuminate; southern Ethiopia, Somalia and further south, 650–1900 m elevation	**97**
97a	Leaf base cuneate to acuminate; anthers 3.5–4 mm long; stem trichomes with midpoints 0.4–1(2) mm long; Democratic Republic of the Congo, Rwanda, Uganda, Tanzania; rare	***Solanumwittei* Robyns**
97b	Leaf base rounded, rarely cuneate; anthers 4–6 mm long; stem trichomes with midpoints 0.05–0.2 mm long; widespread, common	***Solanumtettense* Klotzsch**
98a	Plants with no prickles visible	**99**
98b	Plants with at least some visible prickles.	**109**
99a	Leaves 3–8 times longer than wide, narrow-elliptic or lanceolate, rarely narrow-ovate	**100**
99b	Leaves 1–3 times longer than wide, orbicular, ovate, obovate, elliptic or oblong	**101**
100a	Corolla 0.9–1.5 cm in diameter; trichomes on young stems with ca. 8 rays; native plant in eastern and north-eastern Africa, black cotton soils	***Solanumlanzae* J.-P.LeBrun & Stork**
100b	Corolla 2.5–3(5) cm in diameter; trichomes on young stems with 10–14(16) rays; weed in southern Africa and Mediterranean	***Solanumelaeagnifolium* Cav.**
101a	Leaves less than 1.5 times longer than wide, orbicular, ovate or obovate; arid north-eastern Africa	**102**
101b	Leaves more than 1.5 times longer than wide, ovate, elliptic or oblong; widespread	**103**
102a	Erect shrub to small tree 1.5–4 m tall; leaves and flowers readily deciduous, so stems often bare; seeds orange-brown	***Solanumjubae* Bitter**
102a	Scandent to erect shrub up to 1 m tall; leaves and flowers not readily deciduous, stems not bare; seeds dark brown to almost black	***Solanumcordatum* Forssk.**
103a	Leaf base attenuate; corolla lobed for ca. 3/4 of the way to the base; trichomes on the young stems with 10–20 rays	**104**
103a	Leaf base cuneate, rounded or truncate, rarely attenuate; corolla lobed for 1/2–2/3 of the way to the base; trichomes on the young stems with 6–9(12) rays	**105**
104a	Petioles 1/3–2/3 as long as the adult leaves; flowers 4–8 per inflorescence; seeds 2.8–3.5 mm long; Angola	***Solanumpauperum* C.H.Wright**
104b	Petioles 1/6–1/4 as long as the adult leaves; flowers 8–15 per inflorescence; seeds 3.6–4.8 mm long; eastern and south-eastern Africa	***Solanumgoetzei* Dammer**
105a	Calyx lobes 5–10 mm long in flower, foliaceous, ovate to elliptic; leaf apex rounded, sometimes obtuse; arid north-eastern Africa	***Solanumpampaninii* Chiov.**
105b	Calyx lobes 1–4.5 mm long in flower, not clearly foliaceous, deltate; leaf apex acute or obtuse, sometimes rounded; widespread	**106**
106a	Scandent shrub; inflorescences 3.5–7 cm long; young stems with stalked trichomes, the stalks 0.1–0.4 mm long; eastern African highlands	***Solanumstipitatostellatum* Dammer**
106b	Erect herb or shrub; inflorescences 1–3.5 cm long; young stems with sessile or stalked trichomes, the stalks up to 0.15 mm long; widespread	**107**
107a	Fruit (1)1.5–2.5(5) cm in diameter; plant cultivated for leaves or fruits; widespread and common in western and eastern Africa	***Solanumaethiopicum* L.**
107b	Fruit (0.7)0.75–1.1 cm in diameter; wild plant in southern Africa	**108**
108a	Leaves elliptic; corolla 1.5–2.2 cm in diameter, mauve to purple	***Solanumburchellii* Dunal**
108b	Leaves ovate, sometimes oblong; corolla 0.9–1.3 cm in diameter, usually white	***Solanumcatombelense* Peyr.**
109a	Prickles on young stems predominantly curved	**110**
109b	Prickles on young stems predominantly straight, sometimes straight and reflexed	**125**
110a	Most leaves on reproductive branches less than two times longer than wide	**111**
110b	Most leaves on reproductive branches two times longer than wide or longer	**115**
111a	Corolla lobed for ca. 3/4 of the way to the base or deeper; seeds dark brown to almost black; continental Africa north of the equator	**112**
111b	Corolla lobed for ca. 2/3 of the way to the base; seeds yellow to orange-brown; continental Africa south of the equator	**113**
112a	Inflorescences with 1(2) flowers; trichomes on young stem with 12–18 rays	***Solanumcordatum* Forssk.**
112b	Inflorescences with (1)2–20 flowers; trichomes on young stem with 6–10 rays	***Solanumforskalii* Dunal**
113a	Leaves drying yellowish green; anthers 3–4 mm long; southern Africa	***Solanumlitoraneum* A.E.Gonç.**
113b	Leaves drying reddish brown; anthers (4.5)5.5–8 mm long; Madagascar	**114**
114a	Young stems with a dense covering of long-stalked trichomes and prickles of different lengths; 1000–3000 m elevation	***Solanummyoxotrichum* Baker**
114b	Young stems with a sparse or dense covering of sessile or short-stalked trichomes of uniform lengths; 0–1000 m elevation	***Solanumerythracanthum* Dunal**
115a	Plant erect, 0.4–1.5 m tall; corolla 0.9–1.4 cm in diameter; anthers 2.5–3.5 mm long	**116**
115b	Plant scandent, sometimes erect; corolla (1.2)1.5–3 cm in diameter; anthers 4–8 mm long	**117**
116a	Inflorescence 3–4(6) cm long; trichomes on abaxial surfaces of the leaves with stalks 0.15–0.4 mm long; eastern Africa	***Solanummauense* Bitter**
116b	Inflorescence 1–2.5 cm long; trichomes on abaxial surfaces of the leaves with stalks 0–0.15 mm long; southern Africa	***Solanumcatombelense* Peyr.**
117a	Calyx 7–9 mm long at anthesis, the lobes caudate; trichomes on young stems with midpoints (0.5)1.5–2.5(3) mm long; Tanzanian highlands	***Solanuminaequiradians* Bitter**
117b	Calyx 2–6 mm long at anthesis, the lobes acute to acuminate; trichomes on young stems with midpoints 0–1.5 mm long; widespread	**118**
118a	Leaves 3–6 times longer than wide, the bases attenuate; arid eastern and north-eastern Africa	***Solanumglabratum* Dunal**
118b	Leaves 2–3 times longer than wide, the bases cuneate to cordate; eastern Africa and Madagascar	**119**
119a	Corolla (1.8)2–3 cm in diameter; anthers (5.5)6–10 mm long	**120**
119b	Corolla 1–2(2.4) cm in diameter; anthers 4–6(7) mm long	**122**
120a	Inflorescences with 1–2(5) flowers; young stems with trichomes sessile or stalked, the stalks up to 0.2 mm long; Madagascar	***Solanumerythracanthum* Dunal**
120b	Inflorescences with 3–10 flowers; young stems with trichomes always stalked, the stalks 0.1–0.4 mm long; eastern Africa	**121**
121a	Leaves 3–8 cm long, densely stellate-pubescent on both sides; eastern African coasts	***Solanumusaramense* Dammer**
121b	Leaves 6–13 cm long, adaxially glabrescent; eastern African mountains	***Solanumstipitatostellatum* Dammer**
122a	Leaves apically rounded, with thick-stalked trichomes on the adaxial surface; inflorescence with 1–2(3) flowers; eastern Africa	***Solanumtaitense* Vatke**
122a	Leaves apically acute to obtuse, sometimes rounded, without thick-stalked trichomes on the adaxial surface; inflorescence with (1)2–20 flowers	**123**
123a	Leaves with 2–3 pairs of primary veins; seeds dark brown to almost black; continental Africa north of the equator	***Solanumforskalii* Dunal**
123b	Leaves with 4–6 pairs of primary veins; seeds yellow to orange-brown; continental Africa south of the equator or near the equator	**124**
124a	Prickles usually under 2 mm long; leaves 2.5–5.5 cm long; trichomes on the leaves and stems with stalks ca. 0.1 mm long; inland eastern Africa	***Solanumcyaneopurpureum* De Wild.**
124b	Prickles over 2 mm long; leaves 3–14 cm long; trichomes on the leaves and stems sessile or with stalks under 0.1 mm long; coastal eastern Africa	***Solanumzanzibarense* Vatke**
125a	Leaves more than two times longer than wide	**126**
125b	Leaves less than two times longer than wide	**130**
126a	Corolla 2.5–3(5) cm in diameter; anthers 6–10 mm long; invasive in southern Africa and around the Mediterranean	***Solanumelaeagnifolium* Cav.**
126b	Corolla 0.9–2.3 cm in diameter; anthers 2.5–6 mm long; Ethiopia, Tanzania or further south	**127**
127a	Prickles on young stems 8–16 mm long; calyx 7–12 mm long at anthesis; Ethiopia	***Solanummacracanthum* A.Rich.**
127b	Prickles on young stems 1–6 mm long; calyx 2.5–7 mm long at anthesis; Tanzania or further south	**128**
128a	Plant scrambling shrub; leaves almost glabrous, trichomes on the leaves with up to 4 rays; Tanzanian coastal forest, rare (possibly extinct)	***Solanumruvu* Voronts.**
128b	Plant erect; leaves stellate-pubescent, trichomes on the abaxial sides of the leaves with 17–15 rays; southern Africa	**129**
129a	Leaves elliptic; corolla 1.5–2.2 cm in diameter, mauve to purple	***Solanumburchellii* Dunal**
129b	Leaves ovate, sometimes oblong; corolla 0.9–1.3 cm in diameter, usually white	***Solanumcatombelense* Peyr.**
130a	Leaves on reproductive branches less than 2 cm long	**131**
130b	Leaves on reproductive branches more than 2 cm long	**134**
131a	Leaves and flowers predominantly on short shoots, few on main stems; corolla lobed for 1/2–2/3 of the way to the base; seeds yellow to orange-brown; southern Madagascar	**132**
131b	Leaves and flowers predominantly on main stems, few on short shoots; corolla lobed for ca. 3/4 of the way to the base; seeds dark brown to almost black; continental Africa north of the equator	**133**
132a	Calyx lobes fused in fruit, ca. 10 mm long, fully covering the mature fruit; leaves ovate	***Solanumtoliaraea* D’Arcy & Rakot.**
132b	Calyx lobes free in fruit, 4–5 mm long, the mature fruit at least partly exposed; leaves orbicular, elliptic or ovate	***Solanumbatoides* D’Arcy & Rakot.**
133a	Inflorescences with 1(2) flowers; trichomes on young stem with 12–18 rays	***Solanumcordatum* Forssk.**
133b	Inflorescences with (1)2–20 flowers; trichomes on young stem with 6–10 rays	***Solanumforskalii* Dunal**
134a	Leaves elliptic	**135**
134a	Leaves ovate	**136**
135a	Leaves drying yellow-green; anthers 3.5–5.2 mm long; southern Africa	***Solanumburchellii* Dunal**
135b	Leaves drying reddish brown; anthers 5.5–8 mm long; Madagascar	***Solanumerythracanthum* Dunal**
136a	Anthers 3.2–3.5 mm long; southern Africa	***Solanumtomentosum* L.**
136b	Anthers 4.5–8 mm long; widespread	**137**
137a	Calyx lobes 3–7 mm long at anthesis	**138**
137b	Calyx lobes 0.5–3 mm long at anthesis	**139**
138a	Leaves yellow-green; trichomes on the abaxial sides of the leaves with midpoints 1–2 mm long; Tanzania	***Solanuminaequiradians* Bitter**
138b	Leaves reddish; trichomes on the abaxial sides of the leaves with midpoints to 0.5 mm long; Madagascar	***Solanummyoxotrichum* Baker**
139a	Leaves 6–13 cm long; prickles on young stems 1–2 mm long; highland eastern Africa	***Solanumstipitatostellatum* Dammer**
139b	Leaves 1–5(6) cm long; prickles on young stems 2–10 mm long; continental Africa, north of the equator or Madagascar	**140**
140a	Pedicels 0.2–1 mm long; seeds almost black; continental Africa, north of the equator	***Solanumforskalii* Dunal**
140b	Pedicels 1.5–2(2.5) cm long; seeds dull yellow to orange-brown; Madagascar	***Solanumerythracanthum* Dunal**

## Supplementary materials

Supplementary material 1 List of *Solanum* species occurring in Africa as defined in this paper Data type: species data https://binary.pensoft.net/file/318085This dataset is made available under the Open Database License (http://opendatacommons.org/licenses/odbl/1.0/). The Open Database License (ODbL) is a license agreement intended to allow users to freely share, modify, and use this Dataset while maintaining this same freedom for others, provided that the original source and author(s) are credited.Sandra Knapp, Maria S. Vorontsova, Tiina Särkinen

Supplementary material 2 Specimens examined by the authors for the preparation of these keys Data type: species data https://binary.pensoft.net/file/318086This dataset is made available under the Open Database License (http://opendatacommons.org/licenses/odbl/1.0/). The Open Database License (ODbL) is a license agreement intended to allow users to freely share, modify, and use this Dataset while maintaining this same freedom for others, provided that the original source and author(s) are credited.Sandra Knapp, Maria S. Vorontsova, Tiina Särkinen
